# Elastic Fibers in the Pathogenesis of Cystic Fibrosis: Mechanisms, Consequences, and Therapeutic Implications

**DOI:** 10.3390/life16091487

**Published:** 2026-09-05

**Authors:** Jerome Cantor

**Affiliations:** School of Pharmacy and Allied Health Sciences, St John’s University, Queens, NY 11439, USA; cantorj@stjohns.edu

**Keywords:** cystic fibrosis, elastic fibers, bronchiectasis, pulmonary emphysema, hyaluronan

## Abstract

Cystic fibrosis (CF) is a multisystem autosomal recessive disease caused by mutations in the cystic fibrosis transmembrane conductance regulator (CFTR) gene, affecting approximately 100,000 individuals worldwide. While the primary pathology of CF has historically been framed around airway mucus obstruction, chronic infection, and neutrophilic inflammation, emerging evidence points to profound remodeling of the extracellular matrix (ECM), and elastic fibers in particular, as central contributors to disease progression. Elastic fibers, composed of an elastin core surrounded by a fibrillin-rich microfibril scaffold, provide tissues with the resilience and recoil necessary for repetitive mechanical deformation, properties especially critical in the lung, large airways, skin, and vasculature. In CF, a convergence of proteolytic imbalance, oxidative stress, inflammatory mediators, and impaired CFTR-dependent ion transport conspires to degrade and destabilize these fibers at multiple anatomical sites. The consequences include progressive airflow obstruction, bronchiectasis, emphysema-like changes, impaired mucociliary clearance, and systemic connective tissue vulnerabilities. This paper provides a detailed review of elastic fiber biology, the mechanisms by which CF pathophysiology disrupts elastic fiber integrity, the downstream structural and functional consequences, and potential therapeutic avenues targeting elastic fiber preservation or restoration.

## 1. Introduction

Cystic fibrosis (CF) was first described as a distinct clinical entity in the 1930s by Dorothy Andersen, who characterized a fatal childhood disease marked by pancreatic insufficiency and pulmonary suppuration [1,2]. The underlying genetic defect, mutations in the CFTR gene on chromosome 7q31, was identified in 1989, opening an era of mechanistic investigation into how loss of a chloride-conducting glycoprotein could produce such diverse and severe pathology [3,4,5]. The CFTR protein regulates not only chloride flux but also bicarbonate secretion, sodium absorption through the epithelial sodium channel (ENaC), and the composition of airway surface liquid (ASL), all of which bear on the physical environment in which the extracellular matrix must function [6,7,8,9].

The lung is the principal site of CF-related morbidity and mortality. Progressive pulmonary disease, characterized by recurrent infection with *Pseudomonas aeruginosa*, *Staphylococcus aureus*, and other pathogens, drives an unrelenting cycle of neutrophilic inflammation, proteolysis, and structural remodeling [10]. Bronchiectasis, irreversible dilation and destruction of airways, is the radiological hallmark of advanced CF lung disease and reflects the cumulative failure of the airway wall to maintain its architectural integrity [11].

**Figure 1:**
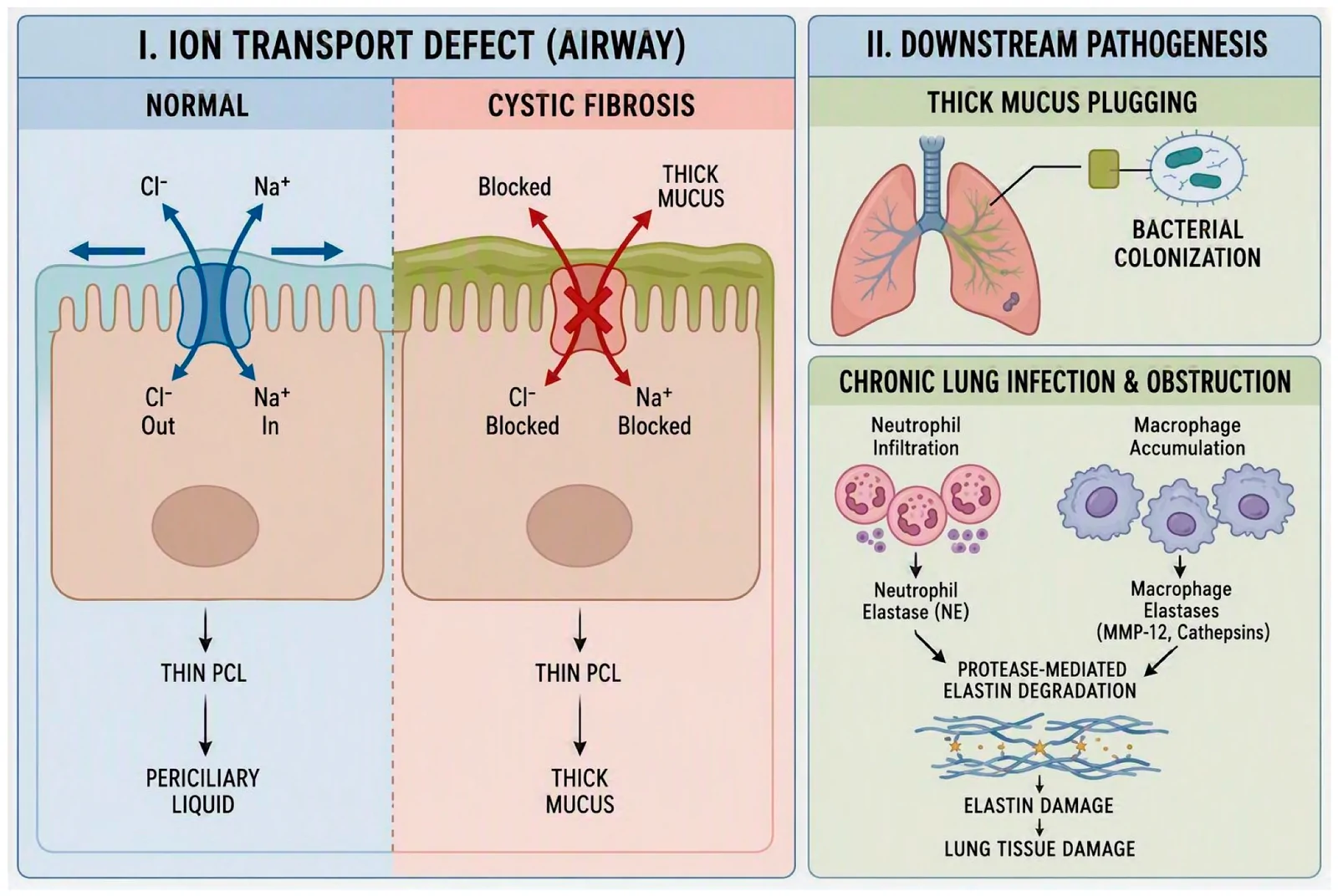
Inflammation due to bacterial colonization of thickened mucus results in the release of neutrophil and macrophage elastases that degrade elastic fibers in the lung airways.

Central to airway wall architecture are elastic fibers, which provide the passive recoil forces that drive expiratory airflow, support alveolar geometry, and maintain airway caliber during tidal breathing [12]. Elastic fiber degradation has been documented in the lungs of CF patients since early morphological studies, and subsequent biochemical investigations have confirmed that elastolytic enzymes, chiefly neutrophil elastase (NE) and matrix metalloproteinases (MMPs), are markedly overrepresented in CF airways (Figure 1) [13,14,15]. However, the biology of elastic fiber pathology in CF extends well beyond simple enzymatic cleavage. Glycation, oxidation, crosslink disruption, and impaired biosynthetic repair all contribute to a net deficit of functional elastic tissue [16,17].

This narrative review synthesizes current knowledge across these domains. We begin with an overview of elastic fiber structure and function, proceed to a systematic analysis of the mechanisms by which CF disrupts lung elastic fiber homeostasis, and conclude with a discussion of emerging therapeutic strategies that may preserve or restore elastic fiber integrity. The specific procedures used to prepare this study are summarized in Table 1.

## 2. Elastic Fiber Biology: Structure, Assembly, and Function

### 2.1. Composition and Hierarchical Organization

Elastic fibers are complex macromolecular assemblies whose functional properties emerge from a highly ordered molecular architecture [12]. The mature fiber consists of a centrally deposited, crosslinked elastin core surrounded and organized by a sheath of fibrillin-1 and fibrillin-2 microfibrils, along with a suite of associated glycoproteins including fibulins (particularly fibulin-4 and fibulin-5), microfibril-associated glycoproteins (MAGPs), and latent TGF-β binding proteins (LTBPs) [18].

**Figure 2:**
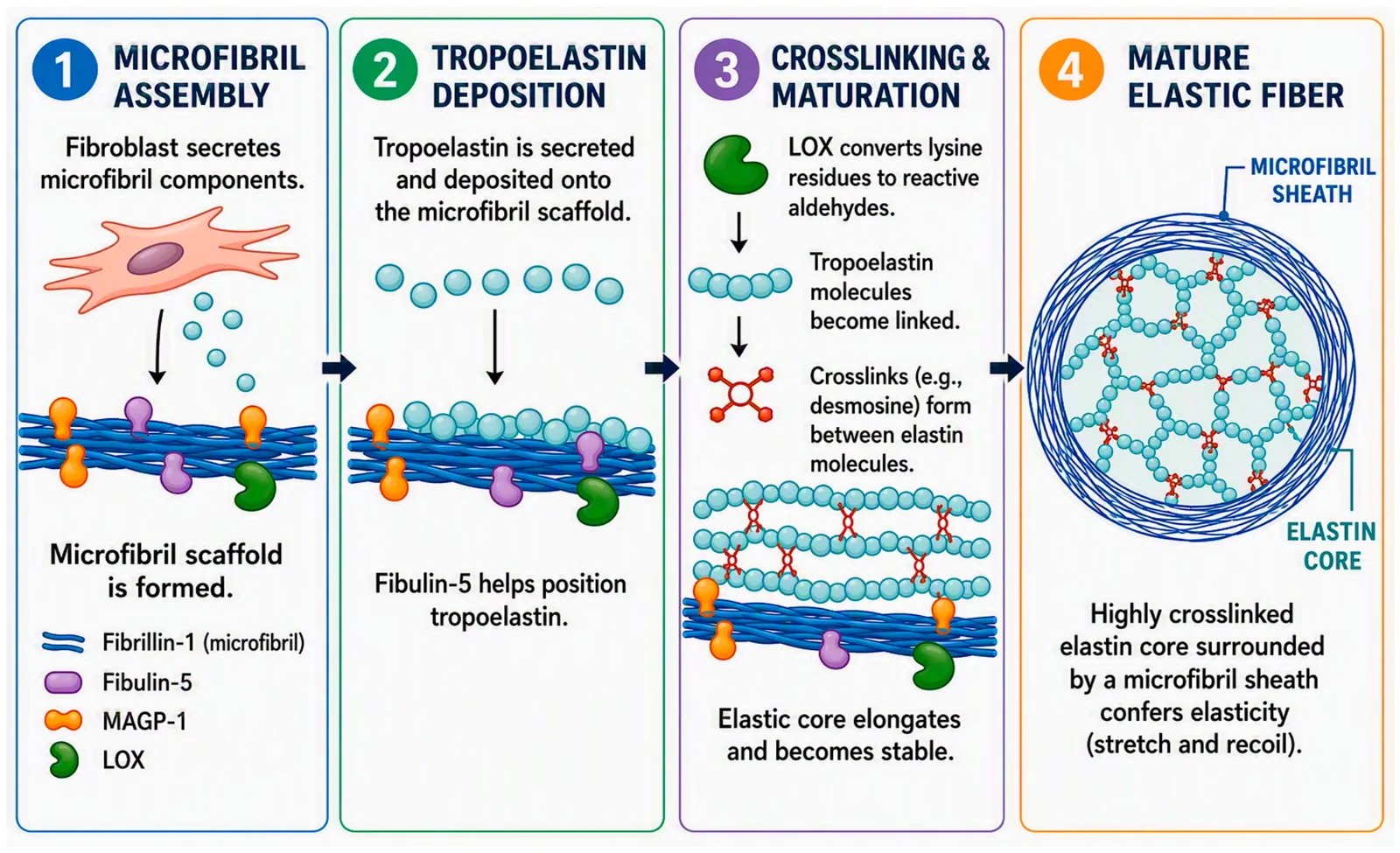
Elastic fiber formation is a complex process involving the integration of a densely crosslinked elastin core with surrounding microfibrils. Mature elastic fibers may have a very long life-span unless exposed to neutrophil and macrophage elastases.

Elastin itself is derived from the soluble precursor tropoelastin, a 72 kDa protein encoded by a single gene (ELN) on chromosome 7q11 [19]. Tropoelastin is an unusual protein in that it contains alternating hydrophobic and crosslinking domains. The hydrophobic domains, rich in valine, proline, glycine, and alanine, undergo a temperature-dependent coacervation that drives phase separation and fiber coalescence, while the crosslinking domains contain lysine residues that are oxidatively deaminated by lysyl oxidase (LOX) to form allysine residues, which then undergo spontaneous condensation to form desmosine and isodesmosine crosslinks that are unique to elastin and responsible for its extraordinary durability [19]. A single tropoelastin molecule may participate in hundreds of such crosslinks within the mature fiber, yielding a macroscopic material with a half-life measured in decades (Figure 2) [16].

The microfibrillar scaffold plays multiple roles: it provides a template upon which tropoelastin is deposited during fiber assembly, it confers tensile strength and mediates interactions with other ECM components, and it serves as a reservoir for growth factors, particularly transforming growth factor-β (TGF-β), whose bioavailability is regulated by mechanical strain and proteolytic events [18]. Tropoelastin itself also interacts directly with cell-surface glycosaminoglycans through its C-terminal domain, a property relevant to how glycosaminoglycan-based therapeutics such as hyaluronan (HA) might interact with elastic fibers [20].

### 2.2. Elastic Fiber Assembly

Elastic fiber assembly is a complex, cell-directed process that proceeds through several stages: (1) intracellular synthesis and secretion of tropoelastin, (2) cell-surface directed coacervation mediated by elastin-binding protein (EBP), (3) deposition onto pre-existing microfibrillar templates, and (4) crosslinking by LOX family enzymes [18,19,21]. This assembly occurs primarily during fetal and early postnatal development. Adult tissues have limited capacity for elastic fiber neosynthesis, making degradation essentially irreversible under most pathological conditions [12].

MAGP-1 and MAGP-2 facilitate tropoelastin deposition onto microfibrils, while fibulin-4 and fibulin-5 serve as molecular bridges that connect tropoelastin to the fibrillin scaffold and present tropoelastin to LOX for crosslinking [18]. Fibulin-5 in particular is essential for elastic fiber assembly in vivo, and fibulin-5-null mice exhibit severe emphysema, cutis laxa, and vascular abnormalities that closely parallel human disease [22,23]. Fibulin-4, a related matricellular protein, similarly directs elastogenesis by activating lysyl oxidase and forming ternary complexes with tropoelastin [24,25].

### 2.3. Tissue-Specific Functions

The mechanical requirements for elastic fibers differ substantially by tissue. In the lung, elastic fibers in the alveolar walls and large airways provide the passive recoil that drives expiration and prevents overdistension during inspiration, and disruption of this network is the primary mechanical basis of emphysema [12]. In large airways, elastic fibers in the submucosa and adventitia help maintain airway caliber against the transmural pressure gradients generated during cough and forced expiration [12]. In the pulmonary vasculature, elastic fibers in the media of pulmonary arteries dampen the pulsatile flow generated by right ventricular contraction [12].

Beyond their mechanical functions, elastic fibers perform important signaling functions. Their degradation products, specifically elastin-derived peptides (EDPs) containing the VGVAPG motif, bind to the elastin receptor complex and stimulate fibroblast and smooth muscle cell migration, trigger MMP production, and modulate inflammatory cell recruitment [12]. Thus, elastic fiber degradation is not merely a structural loss but generates bioactive fragments that amplify inflammatory and remodeling cascades [18,26,27].

## 3. CFTR Biology and Its Relationship to the Extracellular Matrix

CFTR is a member of the ABC transporter superfamily and functions as a cAMP-regulated chloride and bicarbonate channel expressed at the apical surface of epithelial cells lining airways, intestine, pancreatic ducts, sweat glands, and other secretory epithelia [28,29]. Numerous disease-associated variants have been identified in the CFTR gene, classified based on their molecular mechanism: class I (no protein produced), class II (misfolding and degradation—the most common, including F508del), class III (gating defects), class IV (reduced conductance), class V (reduced protein), and class VI (instability at the cell surface) [28].

CFTR trafficking, folding, and surface stability continue to define distinct opportunities for mutation-specific correction [30]. The relationship between CFTR dysfunction and ECM remodeling is multifactorial. CFTR-deficient epithelia produce dehydrated, acidic ASL, which impairs mucociliary clearance, promotes microbial colonization, and creates a proinflammatory environment in which proteolytic enzymes accumulate [28].

Furthermore, the bicarbonate transport function of CFTR is critical for the proper unfolding and secretion of mucins. In its absence, mucus is abnormally compact and adherent, further impairing clearance [31,32]. CFTR has also been shown to modulate intracellular signaling pathways which regulate the production of inflammatory cytokines and MMP expression, and there is growing evidence that CFTR dysfunction directly affects fibroblast and myofibroblast behavior, with consequences for ECM synthesis and remodeling [14,18,33,34].

Particularly relevant is the observation that CFTR-deficient airway epithelial cells show constitutively elevated NF-κB activity, leading to overproduction of IL-8, IL-6, and TNF-α even in the absence of infection [14]. These cytokines directly and indirectly stimulate the production and activation of elastolytic enzymes by recruited neutrophils and macrophages, thereby linking CFTR genotype to elastic fiber vulnerability. However, direct evidence linking specific CFTR mutation classes to the magnitude of pulmonary elastic fiber degradation remains limited. Most mechanistic data derive from comparisons of CF patients as a group with non-CF controls, rather than from genotype-stratified analyses of elastic fiber loss or turnover.

## 4. Mechanisms of Elastic Fiber Degradation in Cystic Fibrosis

### 4.1. Neutrophil Elastase

Neutrophil elastase (NE) is the most destructive single protease in the CF airway, and its chronic dysregulation is central to the broader inflammatory phenotype of CF airway disease [35,36]. Secreted by the abundant neutrophils that characterize CF airway inflammation, NE is a serine protease with potent elastolytic activity that cleaves multiple ECM substrates, including elastin, fibronectin, laminin, and collagen [14]. Under normal circumstances, NE is rapidly inhibited by alpha-1 antitrypsin (AAT) and secretory leukocyte protease inhibitor (SLPI); however, in CF airways, the burden of NE vastly exceeds the capacity of endogenous inhibitors, resulting in net unopposed elastolysis [14,37,38].

The consequences of unrestrained NE activity in CF extend beyond direct ECM cleavage. NE cleaves and inactivates complement receptors on phagocyte surfaces, allowing Pseudomonas to persist despite an intense inflammatory response [14]. NE also cleaves CFTR itself, potentially worsening chloride transport dysfunction, and inactivates antimicrobial peptides, including defensins and lactoferrin, further compromising innate immunity [39]. Free NE in CF bronchoalveolar lavage fluid (BALF) is detectable from infancy and correlates with lung function decline, making it both a biomarker and a mechanistic driver of structural injury [15,40,41].

**Figure 3:**
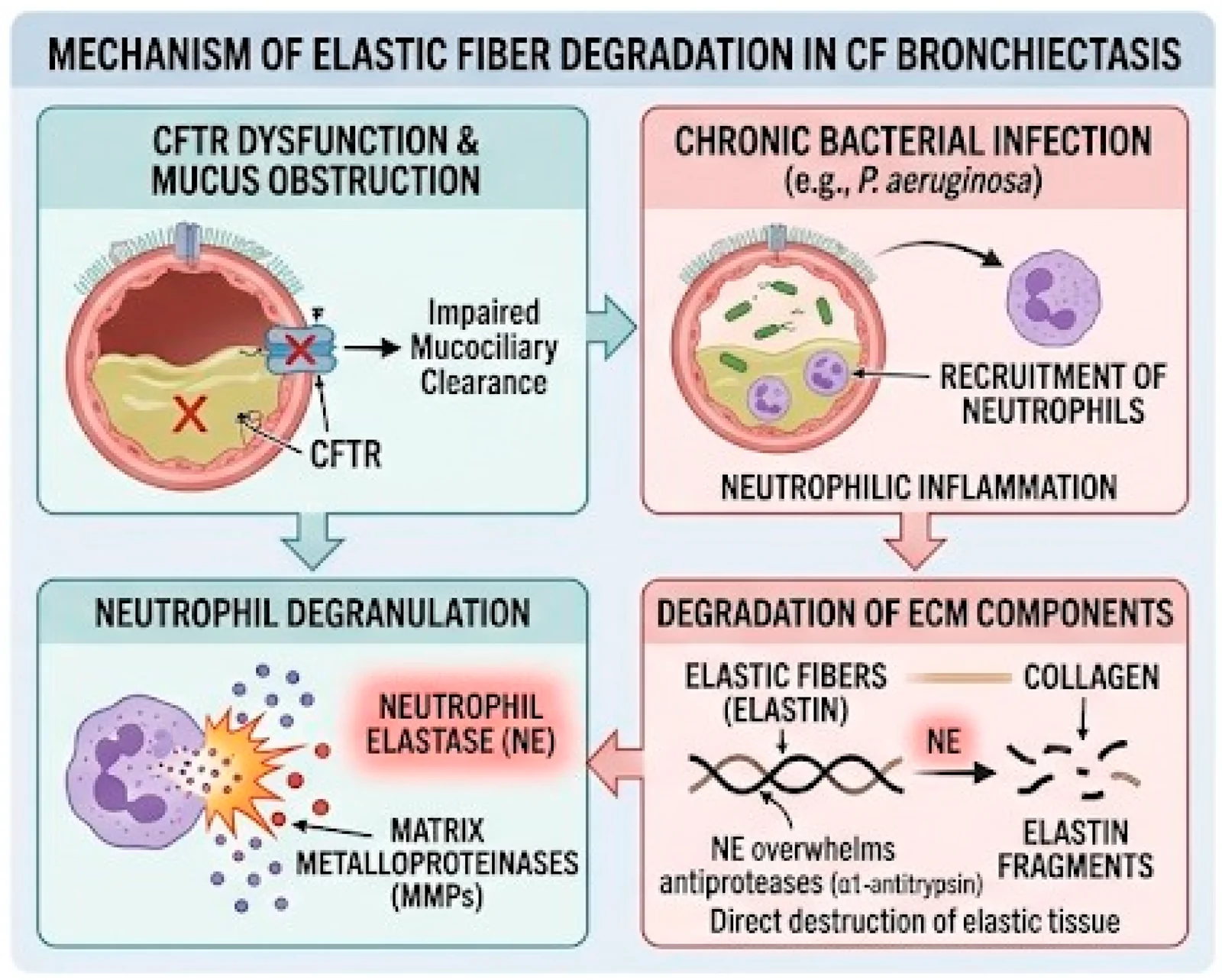
Bacterial infection-induced neutrophil and macrophage recruitment is associated with elastic fiber degradation in bronchial walls and EDP-induced inflammation, leading to structural changes associated with bronchiectasis.

Quantitative analyses have demonstrated that NE concentrations in CF BALF routinely exceed 100 nM, a level at which free enzymatic activity substantially outpaces the neutralizing capacity of endogenous antiproteases such as AAT [14]. Longitudinal studies have confirmed that early NE burden predicts subsequent structural lung disease on CT imaging, supporting an association between NE-mediated elastolysis and bronchiectasis, although this relationship is based on observational and correlative data rather than direct mechanistic evidence (Figure 3) [13].

### 4.2. Matrix Metalloproteinases

Matrix metalloproteinases (MMPs) are zinc-dependent endopeptidases that collectively can degrade all ECM components. In CF airways, MMP-2, MMP-8, MMP-9, and MMP-12 are significantly elevated compared to healthy controls or individuals with other chronic lung diseases [14,42]. MMP-9, principally secreted by neutrophils and macrophages, has potent elastolytic and gelatinolytic activity and is present at markedly elevated concentrations in CF sputum and BALF [15]. MMP-12, produced by macrophages, is a dedicated elastase whose expression is upregulated by tobacco smoke, bacterial products, and the inflammatory milieu of CF airways [15,43].

The MMP/TIMP (tissue inhibitor of metalloproteinases) ratio is critically disrupted in CF. While TIMP-1 levels are elevated in CF airways, this elevation is insufficient to compensate for the massive MMP overproduction, resulting in a net proteolytic environment [15,44,45]. Moreover, NE can activate latent pro-MMPs and degrade TIMPs, creating synergistic elastolytic activity between serine and metalloprotease systems [15,46]. This protease cascade amplifies elastic fiber degradation far beyond what either enzyme class could achieve alone.

MMP-derived elastin fragments, like those generated by NE, are not biologically inert. The EDPs generated by MMP cleavage activate macrophages to produce IL-1β and TNF-α, stimulate fibroblast proliferation, and promote smooth muscle cell migration, all of which contribute to airway wall remodeling and the progressive distortion of bronchial architecture seen in CF [12,47].

### 4.3. Cathepsins

Cysteine cathepsins, particularly cathepsins B, K, L, and S, represent a third class of elastolytic proteases relevant to CF pathology. Cathepsin S is expressed by airway epithelial cells, macrophages, and dendritic cells, and its expression is upregulated in response to the proinflammatory environment of CF airways [12]. It efficiently cleaves elastin, fibronectin, and laminin and has been shown to activate MMP-9 and degrade SLPI, thereby indirectly amplifying NE and MMP activity [15]. Elevated cathepsin activity has been documented in CF sputum, and cathepsin S levels correlate inversely with lung function parameters, including FEV_1_ [15].

The lysosomal compartment dysfunction associated with CFTR absence—characterized by lysosomal alkalinization and impaired autophagy—may further dysregulate cathepsin activity, as these enzymes are normally processed and activated in the acidic lysosomal environment [48]. This mechanism represents a potentially direct link between CFTR genotype and altered cathepsin-mediated ECM proteolysis.

### 4.4. Oxidative Stress and Elastic Fiber Oxidation

CF airways are characterized by profound oxidative stress, driven by the respiratory burst of accumulated neutrophils, impaired antioxidant defenses, and mitochondrial dysfunction in CFTR-deficient epithelial cells [14]. Reactive oxygen species (ROS)—including superoxide, hydrogen peroxide, and hypochlorous acid generated by myeloperoxidase—directly attack elastic fiber components. Oxidation of methionine, tryptophan, and cysteine residues within fibulin and fibrillin molecules impairs their ability to organize and stabilize the tropoelastin-fibrillin interface [12].

Elastin itself is relatively resistant to direct oxidative cleavage, but oxidation of associated microfibrillar proteins renders the overall elastic fiber more susceptible to proteolytic attack [12]. Hypochlorous acid generated by myeloperoxidase can oxidize methionine residues in AAT (forming AAT-met358-ox), abrogating its anti-NE activity and contributing to the protease-antiprotease imbalance that characterizes CF airways [12]. This oxidative inactivation of antiproteases represents a particularly vicious amplification loop, consistent with the broader oxidant burden documented across CF airway inflammation [35]: inflammation produces ROS, which inactivate protease inhibitors, allowing greater proteolysis, which in turn generates more inflammatory mediators.

Glycation of elastic fiber components represents a distinct chemical modification pathway. Advanced glycation end products (AGEs) form when reducing sugars react non-enzymatically with lysine residues in ECM proteins, forming irreversible crosslinks that stiffen and fragilize tissues [12]. While AGE accumulation is most prominent in diabetes, the chronic airway inflammation and altered mucus composition of CF may create local conditions that accelerate AGE formation within the submucosal ECM. However, direct evidence in CF patients remains to be fully established [49,50,51].

### 4.5. Impaired Elastic Fiber Repair

Given the essentially irreplaceable nature of mature elastic fibers in adults, impairment of biosynthetic repair mechanisms is as important as degradation in determining net elastic fiber content. Tropoelastin synthesis requires a complex set of intracellular and extracellular factors, including adequate copper availability for LOX, appropriate TGF-β and fibulin-5 signaling, and a permissive biomechanical environment [18,52]. In CF, several of these requirements are compromised.

The inflammatory cytokine milieu of CF airways, which is rich in IL-1β, TNF-α, and IL-17, suppresses tropoelastin gene expression in airway fibroblasts while simultaneously stimulating MMP production, creating a biochemical context in which degradation chronically outpaces synthesis [12]. TGF-β, which normally stimulates ECM production, including tropoelastin and fibrillin, is present in CF airways at elevated concentrations. However, its downstream signaling is partly hijacked toward fibrotic pathways (excess collagen and fibronectin deposition) rather than elastic fiber repair, resulting in architectural distortion rather than restoration [53]. This fibrotic remodeling, characterized by subepithelial fibrosis, peribronchial collagen deposition, and airway smooth muscle hypertrophy, progressively replaces the normal elastic fiber scaffold with mechanically inferior fibrous tissue.

## 5. Structural and Functional Consequences of Elastic Fiber Pathology in CF

### 5.1. Bronchiectasis and Airway Wall Remodeling

**Figure 4:**
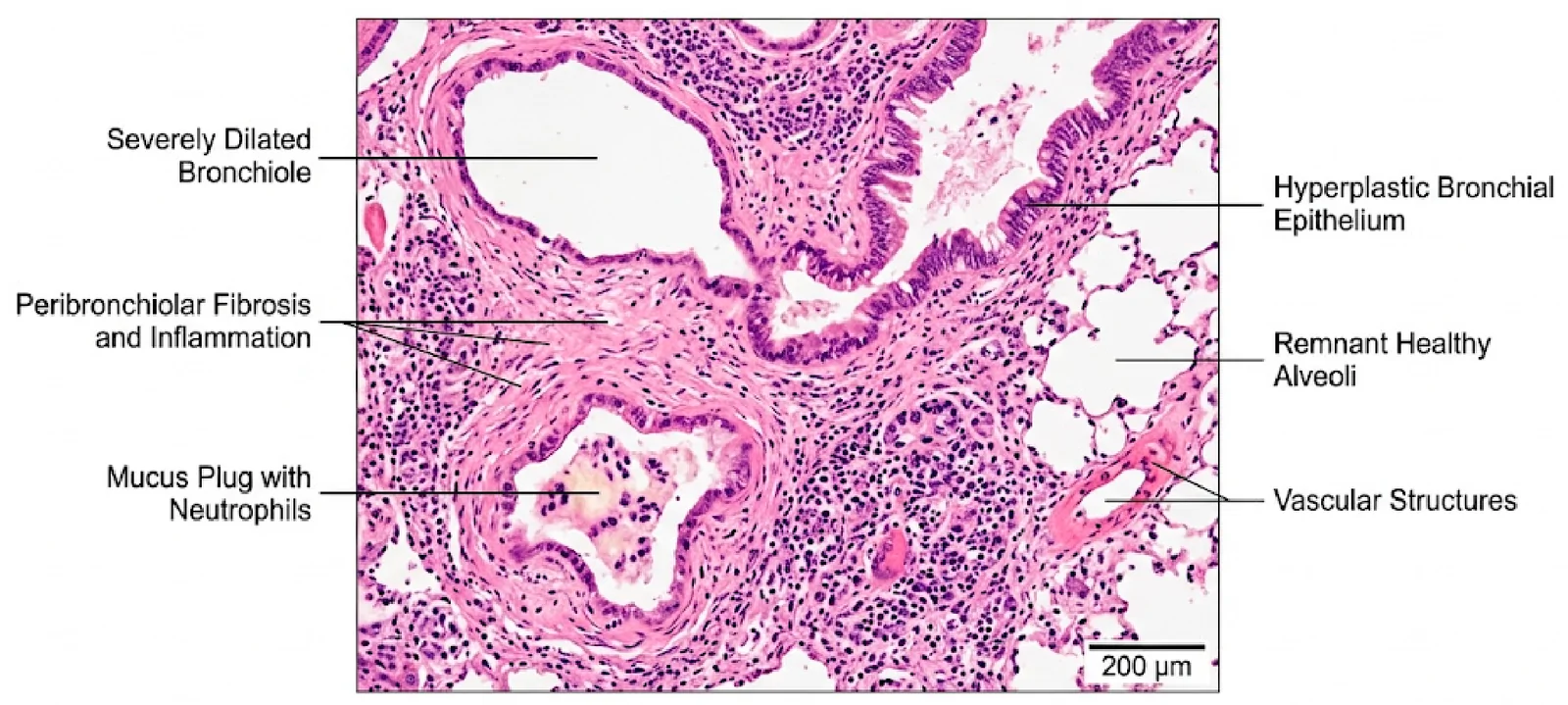
AI-generated micrographic image showing the morphological features of cystic fibrosis-induced bronchiectasis.

Bronchiectasis, the pathological dilation and distortion of bronchi, is the defining structural lesion of progressive CF lung disease (Figure 4) [54]. While mucus obstruction and recurrent infection initiate the process, irreversible airway dilation reflects the failure of the elastic and muscular components of the bronchial wall to maintain circumferential tension. Elastic fibers in the airway adventitia and submucosa provide the restoring forces that prevent progressive dilation; their loss removes a critical mechanical constraint on airway geometry.

Detailed morphometric studies of CF lung specimens at explantation have demonstrated severe depletion of elastic fibers in bronchiectatic segments, with near-complete absence of the normal submucosal elastic network in the most severely affected areas [13]. These changes are accompanied by replacement with disorganized collagen, chronic inflammatory infiltrates, and bronchial wall hypertrophy. The loss of elastic fiber support also contributes to dynamic airway collapse during expiration, which is prevalent in CF patients and further impairs expiratory flow and cough effectiveness.

### 5.2. Emphysema and Alveolar Destruction

**Figure 5:**
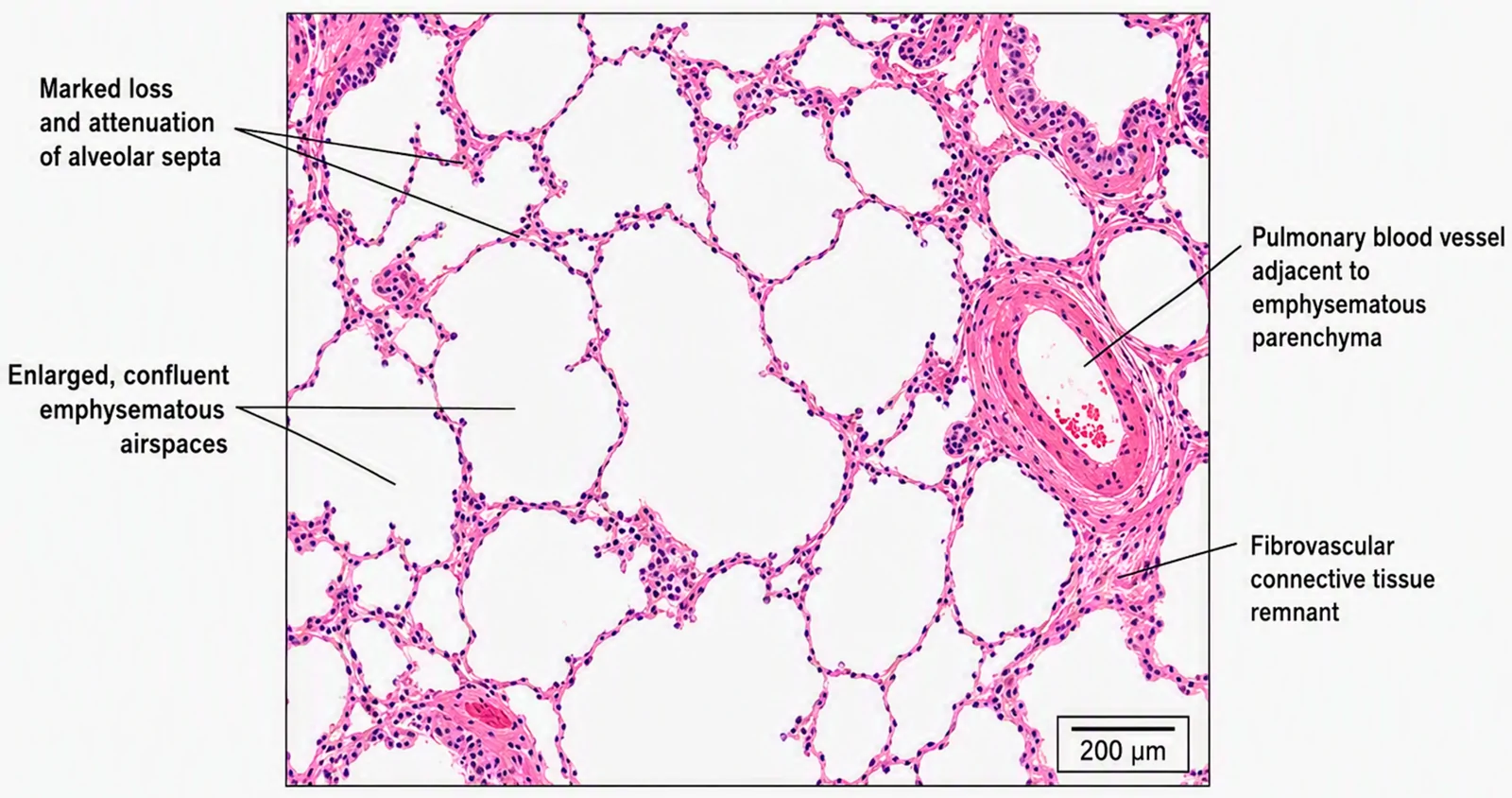
AI-generated micrographic image showing the morphological features of cystic fibrosis-induced pulmonary emphysema.

While classical emphysema is associated with tobacco smoking, a subset of CF patients, particularly those with severe, long-standing disease or with concurrent AAT deficiency, develop emphysematous changes characterized by alveolar wall destruction and loss of elastic recoil (Figure 5). High-resolution CT studies have identified areas of low attenuation, consistent with air trapping and early emphysema, in a significant proportion of CF patients, particularly in the upper lobes, where Pseudomonas colonization is most intense.

**Figure 6:**
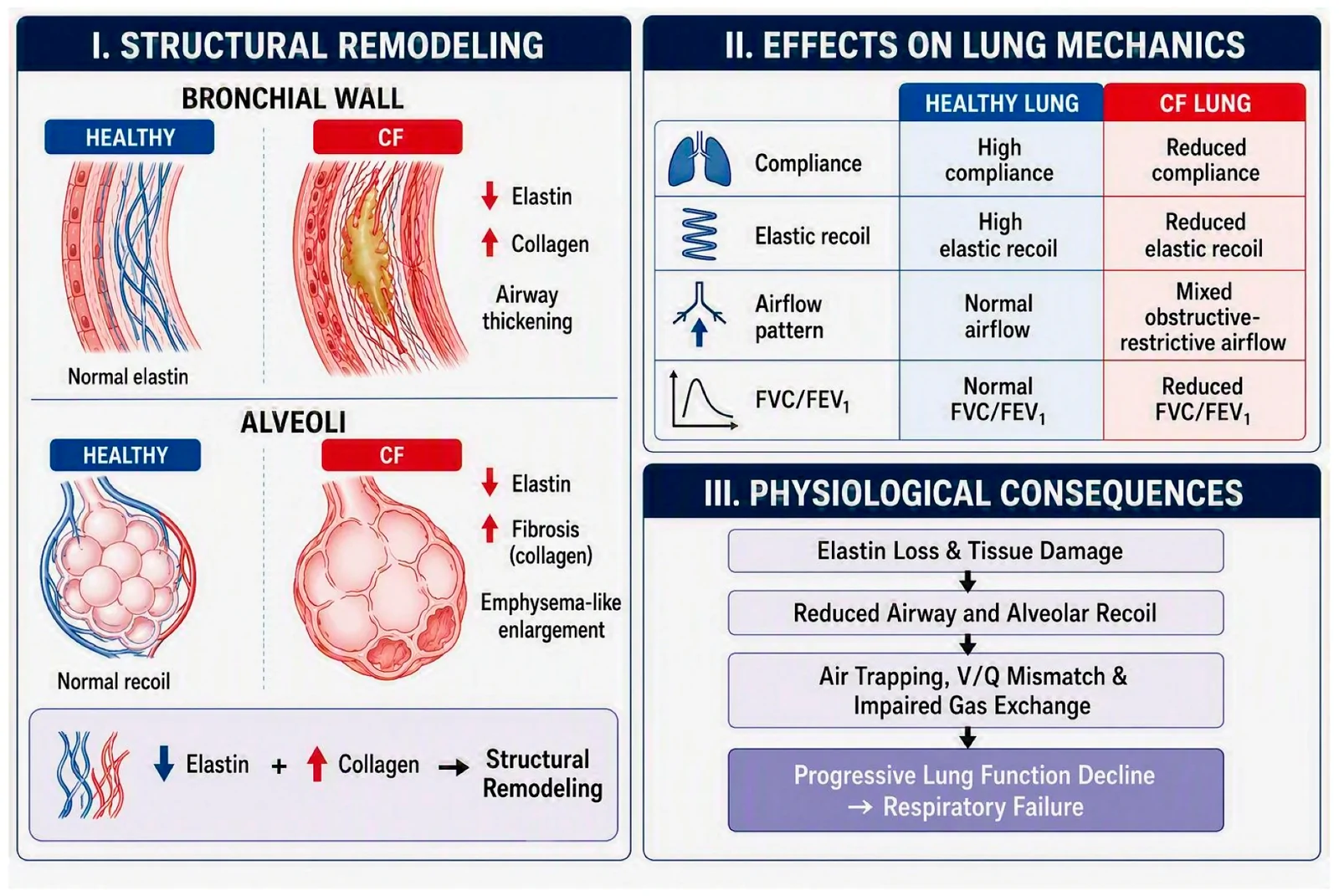
Infection-related recruitment of neutrophils and macrophages is associated with remodeling of the extracellular matrix in bronchial and alveolar walls. causing airway obstruction and emphysema-like airspace enlargement.

The alveolar elastic fiber network is particularly vulnerable in CF because the distal airways and alveoli are sites of the most intense neutrophil degranulation and NE release [48]. The alveolar septum, whose mechanical integrity depends on an intact elastic fiber meshwork spanning the interstitium, becomes progressively fenestrated and disrupted as elastic fiber degradation outpaces repair [14]. Loss of alveolar elastic recoil has direct implications for gas-exchange efficiency and is reflected in a reduction in diffusing capacity (DLCO) seen in advanced CF (Figure 6).

### 5.3. Mucus Clearance and Elastic Fiber Interactions

The mechanical properties of the airway wall—including its compliance and the efficiency of dynamic compression during coughing—are intimately dependent on the integrity of elastic fibers. An effective cough requires that airways compress at appropriate transmural pressures to generate high linear velocities that shear mucus from epithelial surfaces; this compression-recoil cycle depends on the viscoelastic properties of the airway wall. When elastic fibers are depleted or disorganized, the airway wall loses its appropriate compliance, and the cough mechanism becomes less effective, perpetuating mucus retention even in patients who perform chest physiotherapy.

At the molecular level, EDPs generated during CF-related elastolysis have been shown to alter the biophysical properties of mucus directly. They stimulate airway goblet cells and submucosal glands to hypersecrete mucin glycoproteins via binding to the elastin receptor on secretory cells, creating a feed-forward loop in which elastic fiber degradation promotes mucus accumulation, which in turn promotes further infection and inflammation [18]. This connection between matrix biology and mucus biology represents an underappreciated amplification pathway in CF pathophysiology.

### 5.4. Pulmonary Vascular Consequences

Pulmonary hypertension (PH) is an increasingly recognized complication of advanced CF, contributing to right heart dysfunction and reduced exercise tolerance [55]. The pulmonary vasculature depends heavily on elastic fibers in the arterial media for its Windkessel function—the ability to dampen pulsatile flow and reduce right ventricular afterload [12]. In CF, chronic hypoxia secondary to ventilation-perfusion mismatching, combined with the elaboration of vasoconstrictive mediators and direct proteolytic degradation of vascular elastic fibers, contributes to progressive vascular remodeling and PH.

Histological studies of vascular changes in PH have demonstrated fragmentation and loss of elastic laminae in pulmonary arteries, with replacement by collagenous intimal and medial thickening [56]. These changes would impair vascular compliance and increase wave reflection, and further investigation correlating the magnitude of elastic fiber loss in pulmonary vessels with the severity of PH, as measured by echocardiography or right heart catheterization is needed to confirm this relationship.

### 5.5. Extrapulmonary Manifestations

While the lung is the primary site of CF-related elastic fiber pathology, extrapulmonary manifestations deserve attention. The skin of CF patients shows histological abnormalities in elastic fiber morphology, with fragmented and disorganized elastic fibers in the dermis [18]. These changes may contribute to the skin fragility and poor wound healing that are observed clinically and may reflect a systemic effect of the protease/antiprotease imbalance that characterizes CF, as NE and MMP-9 circulate at detectable levels in the blood of patients with severe disease.

The gastrointestinal tract, particularly the intestine and biliary tree, also shows ECM remodeling in CF. Intestinal elastic fibers are important for maintaining the compliance and contractility of the bowel wall, and their disruption may contribute to abnormalities in intestinal motility and an increased risk of meconium ileus seen in CF [57,58]. In the liver, changes in elastic fibers accompany biliary fibrosis (CF-related liver disease), which affects up to 30% of CF patients. However, whether elastic fiber loss is a cause or consequence of hepatic inflammation in this context remains to be fully established [57,59,60].

### 5.6. Clinical Correlates: Symptoms, Exacerbations, and Prognosis

Data directly linking quantified elastic fiber degradation (for example, by desmosine/isodesmosine biomarker levels) to exacerbation frequency or survival in CF are limited. However, studies of urinary and sputum desmosine/isodesmosine have shown that levels rise during pulmonary exacerbations and fall with treatment, and higher circulating levels have been associated with greater CF disease severity, providing indirect evidence for a relationship between ongoing elastolysis and clinical status [61].

These findings support the design of prospective studies formally testing whether desmosine/isodesmosine or other elastin degradation biomarkers independently predict exacerbation frequency or long-term survival, analogous to the established prognostic role of FEV1 decline. The longitudinal evaluation of these biomarkers will become increasingly important given the rapidly changing natural history of CF due to the advent of CFTR modulators.

## 6. Biomarkers of Elastic Fiber Degradation in CF

**Figure 7:**
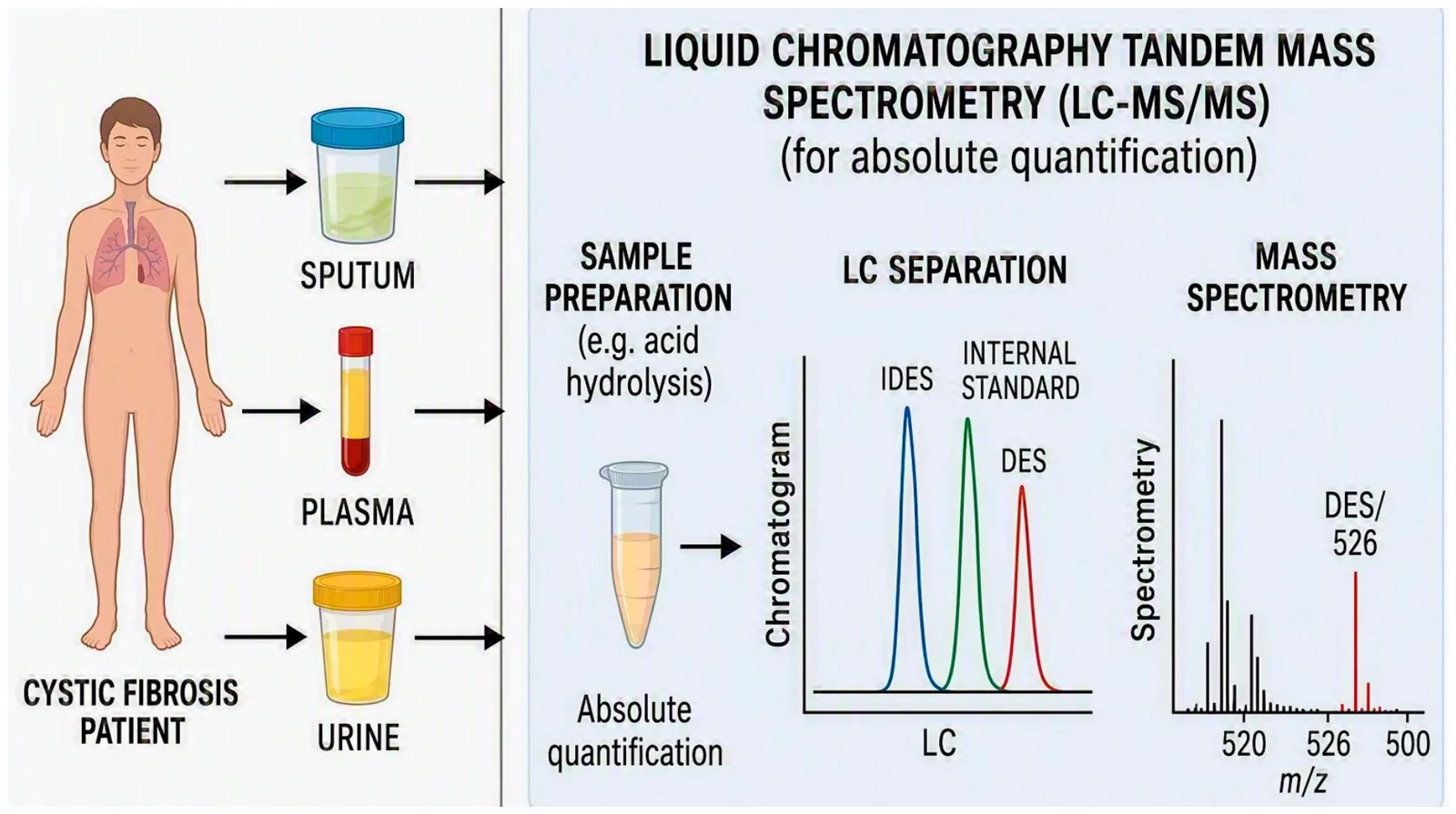
The elastin-specific crosslinks, desmosine and isodesmosine, are elevated in cystic fibrosis and may serve as a biomarker for elastic fiber degradation. Mass spectrometry provides a sensitive and reproducible method of measuring these crosslinks in sputum, plasma, and urine.

Quantifying elastic fiber degradation products in biological fluids provides insight into ECM turnover in CF. Desmosine and isodesmosine (DES/IDE), the unique crosslinks of mature elastin, are released into sputum, BALF, urine, and plasma during elastin catabolism and can be measured by mass spectrometry or ELISA [62,63,64,65]. Urinary DES/IDE levels are significantly elevated in CF patients compared with healthy controls and inversely correlate with FEV_1_, providing a quantitative biomarker of ongoing elastolysis (Figure 7) [61].

Plasma DES/IDE levels, while less robust than urinary measurements due to renal handling effects, show similar trends and have been used in clinical trials of protease inhibitors to confirm target engagement [66]. Sputum DES/IDE levels are markedly elevated in CF and correlate with sputum NE burden, confirming the mechanistic link between NE activity and elastic fiber catabolism [67]. Fibrillin-1 degradation fragments detected in CF BALF provide a complementary marker of microfibrillar component loss and may be particularly relevant given the role of microfibril integrity in elastogenesis.

Other biomarkers of ECM remodeling in CF include circulating fragments of fibronectin, laminin, and collagen crosslinks. While these are not specific to elastic fiber degradation, they collectively reflect the global burden of ECM remodeling and have prognostic relevance in clinical cohort studies. The development of multiplexed biomarker panels incorporating desmosine, elastin peptides, MMP activity markers, and TIMP levels provides a more comprehensive picture of the balance between ECM degradation and repair than any single biomarker can.

Sputum cytology provides a complementary, more accessible window into the cellular processes that drive elastic fiber degradation, although it has been relatively underused as a biomarker in this specific context. Sputum neutrophil counts and percentage correlate broadly with airway NE burden and with the intensity of the inflammatory response that drives elastolysis, and cytological indices of neutrophil activation and degranulation have been proposed as low-cost surrogate measures of the protease burden to which elastic fibers are exposed [68]. However, sputum cytology reflects the overall inflammatory milieu rather than elastic fiber breakdown specifically, and direct correlations between quantitative sputum cytological indices and elastin degradation products such as desmosine/isodesmosine have not been extensively characterized in CF cohorts. Combining cytological assessment with biochemical elastin degradation markers may therefore offer a more complete, and more readily obtainable, picture of ongoing elastic fiber injury than either approach alone, and represents an area warranting further study.

## 7. Modulation of Elastic Fiber Biology by CFTR Modulators

The development of CFTR modulators, small molecules that correct or potentiate CFTR function, has transformed the clinical management of CF and provides important insights into the relationship between CFTR function and elastic fiber biology [69]. Ivacaftor, a CFTR potentiator approved for patients with gating mutations (G551D and others), significantly increases chloride transport and reduces sputum viscosity, thereby improving mucociliary clearance and reducing airway inflammation [70]. Studies of ivacaftor-treated patients have demonstrated reductions in sputum NE activity, IL-8, and MMP-9, suggesting that restoration of CFTR function attenuates the protease burden that drives elastic fiber degradation [71].

The triple combination therapy lumacaftor/tezacaftor/elexacaftor (ETI), approved for patients with at least one F508del allele, produces the most substantial improvements in lung function and quality of life yet seen with CFTR-targeted therapy [72,73]. Early biomarker studies of ETI-treated patients have documented reductions in sputum elastase activity and desmosine levels, consistent with reduced elastic fiber catabolism [74]. However, structural lung disease, particularly established bronchiectasis, does not reverse with CFTR modulator therapy, reflecting the irreversible nature of elastic fiber loss and the limited regenerative capacity of adult elastic fiber networks [75,76]. This observation underscores the importance of early intervention, before structural remodeling becomes irreversible, and highlights the need for adjunctive therapies that target ECM preservation alongside CFTR correction [77].

## 8. Physical Modeling of Pathogenesis: Percolation Theory in Cystic Fibrosis

### 8.1. Percolation Theory

Elastic fiber degradation in CF is not a simple linear function of protease burden but instead reflects the interaction of a heterogeneous, spatially organized network of airways and tissue elements. Percolation theory is introduced here specifically because it offers a quantitative framework for understanding how localized, mucus- and infection-related elastic fiber and airway-network injury can accumulate silently before producing the abrupt, threshold-like clinical deteriorations that are characteristic of CF lung disease.

While cystic fibrosis (CF) originates from single-gene mutations in the cystic fibrosis transmembrane conductance regulator (CFTR) gene, the clinical progression of CF lung disease exhibits non-linear, threshold-driven behavior that standard linear biochemical models fail to fully explain. Percolation theory, a statistical mechanics framework modeling how localized, microscopic connections coalesce into continuous macro-scale networks across critical density thresholds, provides a quantitative framework for these sudden state transitions in CF lung pathology. Rigidity percolation, in particular, has proven a robust tool for predicting discrete material-property transitions in disordered biological networks, ranging from cytoskeletal and embryonic tissue mechanics to macroscale organ behavior, lending physical plausibility to its application in the CF airway [78].

### 8.2. Mucin Gelation and Rigidity Percolation

In healthy airway surface liquid (ASL), secreted mucin polymers (MUC5B and MUC5AC) exist below the critical overlap concentration, behaving as a viscous fluid easily cleared by ciliary beating. Depletion of the ASL volume driven by unchecked Na+ absorption and absent Cl- and HCO_3_ secretion increases local concentrations of mucins, extracellular DNA, and filamentous actin released by necrotic neutrophils, consistent with the periciliary liquid depletion mechanism originally proposed for CF airway surface dehydration [31,32].

As biopolymer density approaches a critical threshold, inter-polymer entanglements and crosslinks undergo a rigidity percolation phase transition. The fluid undergoes a physical shift from a viscous sol to an adherent, solid-like hydrogel matrix, completely arresting mucociliary transport and forming resistant mucus plugs, a transition whose clinical correlate is the abrupt loss of benefit seen when airway hydration therapies are withdrawn or delayed [79,80].

### 8.3. Topological Collapse of the Airway Network

The human tracheobronchial tree operates as a high-dimensional fractal network. Initial mucus plugging causes localized occlusions in distal bronchioles, where gas exchange is temporarily maintained via adjacent collateral pathways. However, as localized occlusions accumulate, the density of blocked branches reaches the structural percolation threshold of the airway lattice. Upon reaching a critical threshold, connected pathways for airflow are abruptly severed across entire lobular or segmental domains. This topological network collapse accounts for the clinical observation wherein patients maintain stable pulmonary function metrics, such as forced expiratory volume in one second, before experiencing sudden, non-linear drops during acute respiratory exacerbations. The process is mechanistically tied to elastic fiber injury in both directions: elastic fiber loss impairs cough-driven mucus clearance, promoting the mucus plugging that drives network occlusion, while the resulting obstructed, inflamed airway segments are themselves sites of intensified elastolytic activity.

### 8.4. Biofilm Hydrogel Microenvironments

Opportunistic pathogens, predominantly *Pseudomonas aeruginosa*, utilize percolation mechanics to establish chronic niches within the stagnant, hypoxic mucus gel. Secreting extracellular polymeric substances (EPS) such as alginate, bacterial microcolonies increase EPS network density until it percolates into a continuous, impenetrable hydrogel mesh surrounding the colony. Crossing this threshold drastically slows the passive diffusion of host immunoglobulins, antimicrobial peptides, and systemic antibiotics, converting vulnerable planktonic bacterial populations into self-sustaining, recalcitrant biofilms, a state that parallels the broader concept of CF as a mucosal immunodeficiency in which the local host-defense network is topologically as well as biochemically compromised [76,81]. This connects directly to elastic fiber pathology: the chronic infection sustained within biofilm niches perpetuates the neutrophil influx and NE/MMP release that drive elastic fiber degradation throughout this review, making biofilm percolation an upstream amplifier of the elastolytic burden.

### 8.5. Extracellular Matrix Remodeling, Elastic Fiber Degradation, and Mechanical Stiffening

Continuous neutrophilic inflammation exposes the pulmonary extracellular matrix (ECM) to massive concentrations of proteolytic enzymes, most notably neutrophil elastase (NE) and matrix metalloproteinases (MMPs such as MMP-8 and MMP-9), consistent with the protease burden characterized biochemically elsewhere in this review [82]. The structural degradation of the lung ECM in cystic fibrosis exhibits a biphasic mechanics shift: early localized loss of elastic compliance followed by an irreversible rigidity percolation transition driven by fibrotic collagen deposition.

In healthy pulmonary tissue, cross-linked elastic fibers (composed of core insoluble elastin draped over fibrillin-microfibril scaffolds) form a continuous network that confers physiological recoil during respiration, whereas unrestrained NE activity in CF cleaves key cross-linking domains in elastin. Electron microscopic and proteomic analyses reveal that the bronchial elastic framework loses its branched architecture, degenerating into fragmented, slender, and lacunar remnants.

The bioactive EDPs produced by proteolytic digestion of elastic fibers, act as signaling molecules that bind to EBP on macrophages, neutrophils, and fibroblasts. EDPs trigger chemotaxis for circulating inflammatory cells and stimulate additional neutrophil degranulation and NE secretion, establishing a destructive, self-sustaining feed-forward signaling loop that is well described for the elastin receptor complex and its matrikine ligands in other chronic inflammatory and remodeling contexts [83,84].

**Figure 8:**
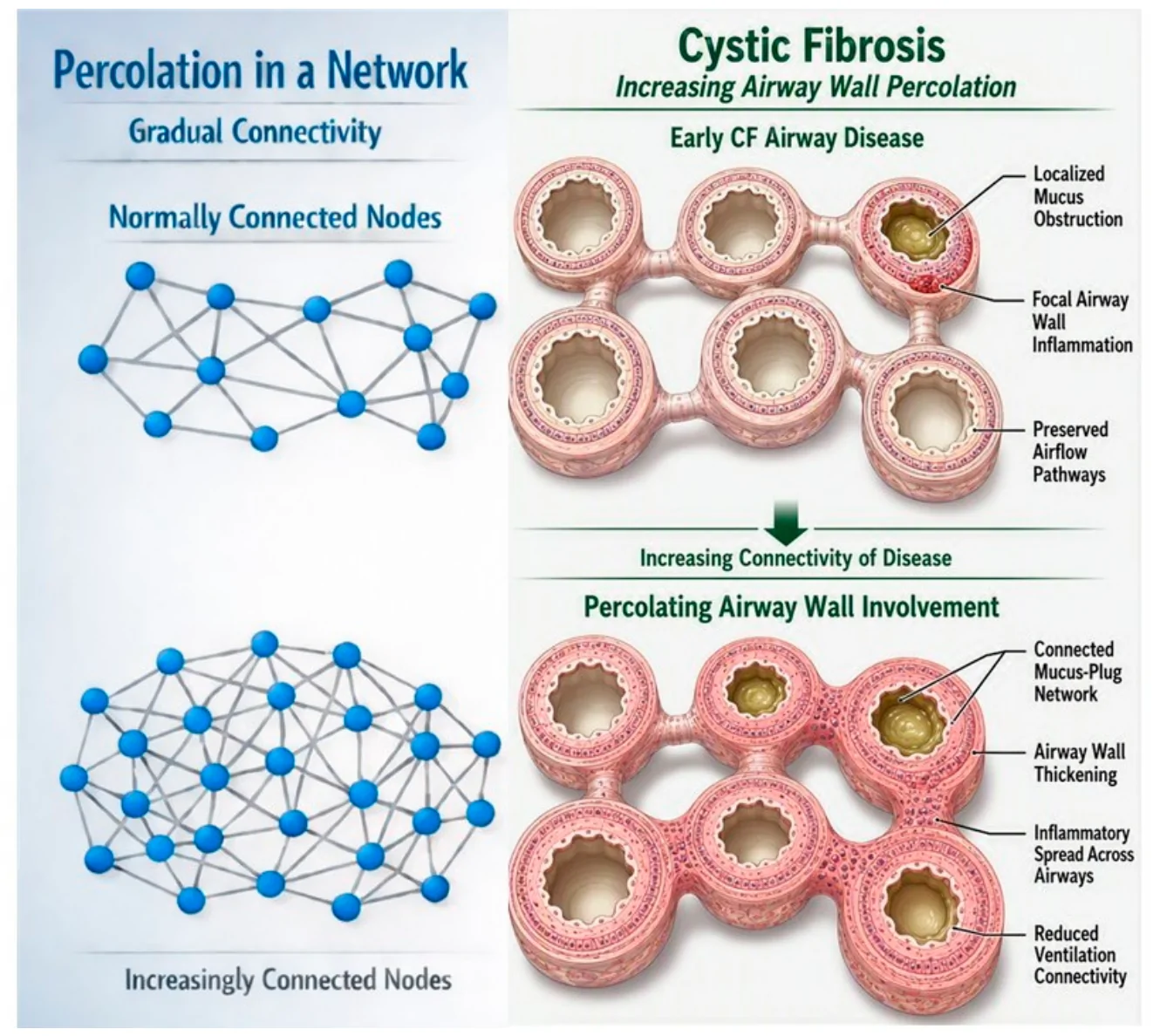
As the fraction of collagen cross-links crosses the rigidity percolation threshold, the mechanical properties of the airway walls undergo a phase transition, characterized by a highly stiffened extracellular matrix. This shift permanently compromises respiratory biomechanics, leading to airway wall destruction and irreversible parenchymal damage characteristic of end-stage bronchiectasis.

Because adult pulmonary tissue lacks significant capacity to regenerate functional elastin, the structural void left by degraded elastic fibers is filled by a disorganized, dense deposition of fibrillar collagens (primarily types I and III). This process can be modeled as a continuum bond-percolation system. As the local volume fraction of rigid collagen cross-links crosses the rigidity percolation threshold while the elastic bond density vanishes, the mechanical properties of the parenchymal wall undergo a discrete phase transition. The dynamic, elastic lung architecture transitions into a non-compliant, highly stiffened matrix. This shift permanently compromises respiratory biomechanics, driving the irreversible parenchymal damage and airway wall destruction characteristic of end-stage bronchiectasis (Figure 8).

## 9. Therapeutic Strategies Targeting Elastic Fiber Preservation in CF

### 9.1. Protease Inhibitor Strategies

The most extensively studied approach to limiting elastic fiber degradation in CF is pharmacological inhibition of NE and MMPs. Inhaled AAT has been evaluated in multiple clinical trials, with evidence of reduced NE activity in sputum and BALF, reductions in inflammatory markers, and modest improvements in some lung function parameters [39,85]. However, robust evidence of efficacy for structural lung disease or hard clinical endpoints remains to be fully established, likely because AAT delivery to peripheral airways is limited and because multiple elastolytic pathways operate simultaneously [86].

SLPI, a serine protease inhibitor with anti-NE and anti-cathepsin activity, has also been investigated as an inhaled agent in CF [14]. DX-890, a potent NE inhibitor derived from elastin-fold domains, showed encouraging effects on NE activity in phase 2 trials [14], and small-molecule NE inhibitors such as AZD9668 have shown comparable pharmacological promise in other neutrophilic airway diseases [87]. The challenge with all inhaled antiprotease strategies is achieving adequate concentrations throughout the heterogeneously diseased CF airway tree, particularly in the mucus-obstructed and structurally distorted airways of patients with advanced disease.

MMP inhibition has been explored experimentally but has not yet advanced to robust clinical trials in CF. Broad-spectrum MMP inhibitors have problematic musculoskeletal side-effect profiles, but selective MMP-12 or MMP-9 inhibitors might offer a more targeted approach to limiting elastic fiber degradation while maintaining acceptable tolerability [15]. The tetracycline antibiotic doxycycline, which inhibits MMP activity independently of its antimicrobial properties, has been studied in small CF cohorts with encouraging effects on sputum MMP-9 levels, and a randomized trial of doxycycline during pulmonary exacerbations reported measurable clinical benefit consistent with this mechanism [88].

### 9.2. Antioxidant Strategies

Given the role of oxidative stress in both direct oxidation of elastic fibers and the inactivation of antiproteases, antioxidant strategies have theoretical appeal for limiting elastic fiber injury in CF. N-acetylcysteine (NAC), a glutathione precursor and direct free-radical scavenger, has been studied extensively in CF, with variable results. In contrast, some studies demonstrate reductions in oxidative stress markers, but consistent improvements in lung function have not been established in larger trials. These findings reflect the difficulty of achieving adequate antioxidant concentrations in the CF airway lumen, which is physically separated from circulating antioxidants by the mucus barrier. Inhaled glutathione supplementation and inhaled NAC have been evaluated in CF, with evidence of reduced oxidative markers in BALF but no definitive clinical benefit in randomized trials [89].

### 9.3. Targeting Elastic Fiber Repair Pathways

Strategies that promote elastic fiber biosynthesis represent a complementary approach to antiprotease and antioxidant interventions. Fibulin-5, which is essential for LOX-mediated tropoelastin crosslinking and elastic fiber assembly, has been investigated as a potential therapeutic target; supplementation with recombinant fibulin-5 in animal models of lung injury promotes elastic fiber repair and reduces emphysema-like changes [18]. The feasibility of fibulin-5 delivery to the CF airway and its stability in the protease-rich CF environment would need to be established.

LOX activity is copper-dependent and can be stimulated by TGF-β signaling; however, as noted above, TGF-β in CF airways tends to drive fibrotic rather than elastogenic repair. Strategies that selectively redirect TGF-β signaling toward elastogenesis (for example, through modulation of SMAD2/3 vs. SMAD1/5/8 pathway balance) might offer a route to promoting elastic fiber repair without exacerbating fibrosis [18]. Emerging RNA-based therapeutic approaches, including mRNA delivery of tropoelastin or LOX, are at preclinical stages but represent a potentially powerful future strategy for augmenting elastic fiber repair capacity in CF lung tissue [90,91].

### 9.4. Gene Therapy and Stem Cell Approaches

**Figure 9:**
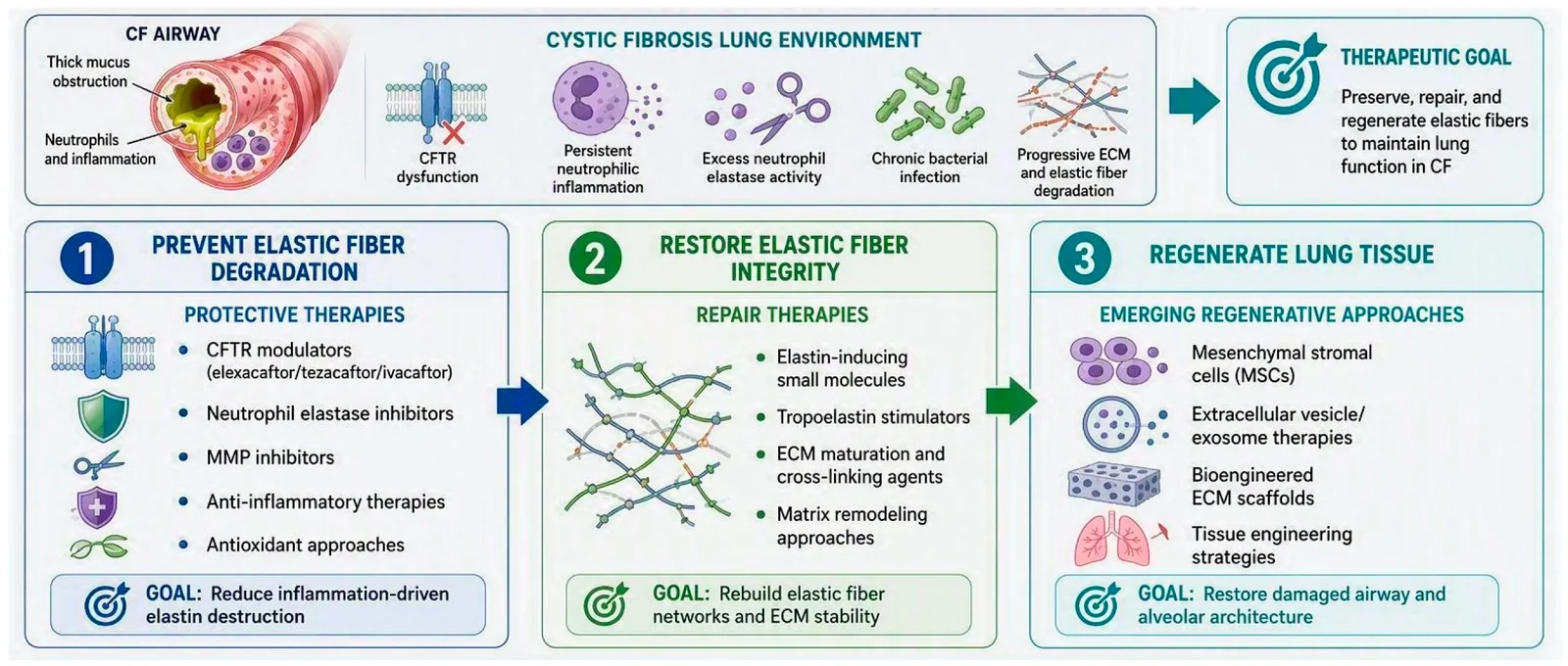
Potential therapies for Cystic Fibrosis include the use of agents that prevent elastic fiber degradation or enhance elastic fiber repair.

Longer-term prospects for elastic fiber restoration in CF may depend on approaches that reconstitute the fibroblast and smooth muscle cell populations responsible for ECM synthesis (Figure 9). Gene-edited autologous stem cells or induced pluripotent stem cells (iPSCs) with corrected CFTR could, in principle, be differentiated into airway mesenchymal cells capable of synthesizing new elastic fiber components; early proof-of-concept studies in organoid models have demonstrated that CFTR-corrected iPSC-derived airway cells could potentially produce a more normal ECM environment than their uncorrected counterparts [92]. The relevance of this approach to elastic fiber preservation specifically lies in its potential to restore normal fibroblast and myofibroblast function, and thereby normal tropoelastin, fibulin, and LOX expression. However, it may not have a direct effect on existing degraded elastic fibers, which are not meaningfully regenerated in the adult lung.

### 9.5. Inhaled HA: A Multiscale Therapeutic Approach

Inhaled HA has emerged as a promising adjunct therapy for cystic fibrosis (CF), a disease characterized by dehydrated airway surfaces, hyperconcentrated mucus, chronic neutrophilic inflammation, and progressive structural damage to the lung. While current inhaled therapies improve mucus clearance, they do not fully correct the biophysical abnormalities of CF mucus or the persistent inflammatory and mechanical stresses that drive airway remodeling. HA’s unique combination of hydrating, viscoelastic, anti-inflammatory, and extracellular matrix–protective properties positions it as a compelling candidate to address these unmet needs [93,94].

HA’s biological behavior depends strongly on molecular weight. High-molecular-weight HA (HMW-HA) provides hydration, lubrication, and anti-inflammatory signaling, whereas low-molecular-weight fragments can be pro-inflammatory. For CF therapy, HMW-HA is the relevant form, since fragmentation of native high-molecular-weight HA into low-molecular-weight species is itself a recognized driver of tissue inflammation [95]. Its ability to bind and retain large volumes of water enables sustained airway hydration, potentially improving ciliary function and reducing mucus concentration more durably than osmotic therapies alone, consistent with HA’s role in regulating fluid retention and shear-dependent transport across epithelial surfaces [96]. HA also interacts with mucins and extracellular DNA, reducing mucus viscosity and adhesivity, key contributors to impaired mucociliary clearance in CF.

A potential, though not yet fully characterized, benefit of HA is a protective effect on elastic fibers, which are essential for airway recoil, airway patency, and effective cough mechanics. In CF, chronic inflammation, particularly neutrophil elastase activity, accelerates elastic fiber degradation, contributing to bronchiectasis and progressive loss of lung elasticity. Evidence for a direct elastic fiber-protective effect of HA comes from in vitro elastase-exposure assays and an elastase-induced airspace enlargement model in rodents, in which HA reduced elastic fiber injury and limited airspace enlargement [97].

**Figure 10:**
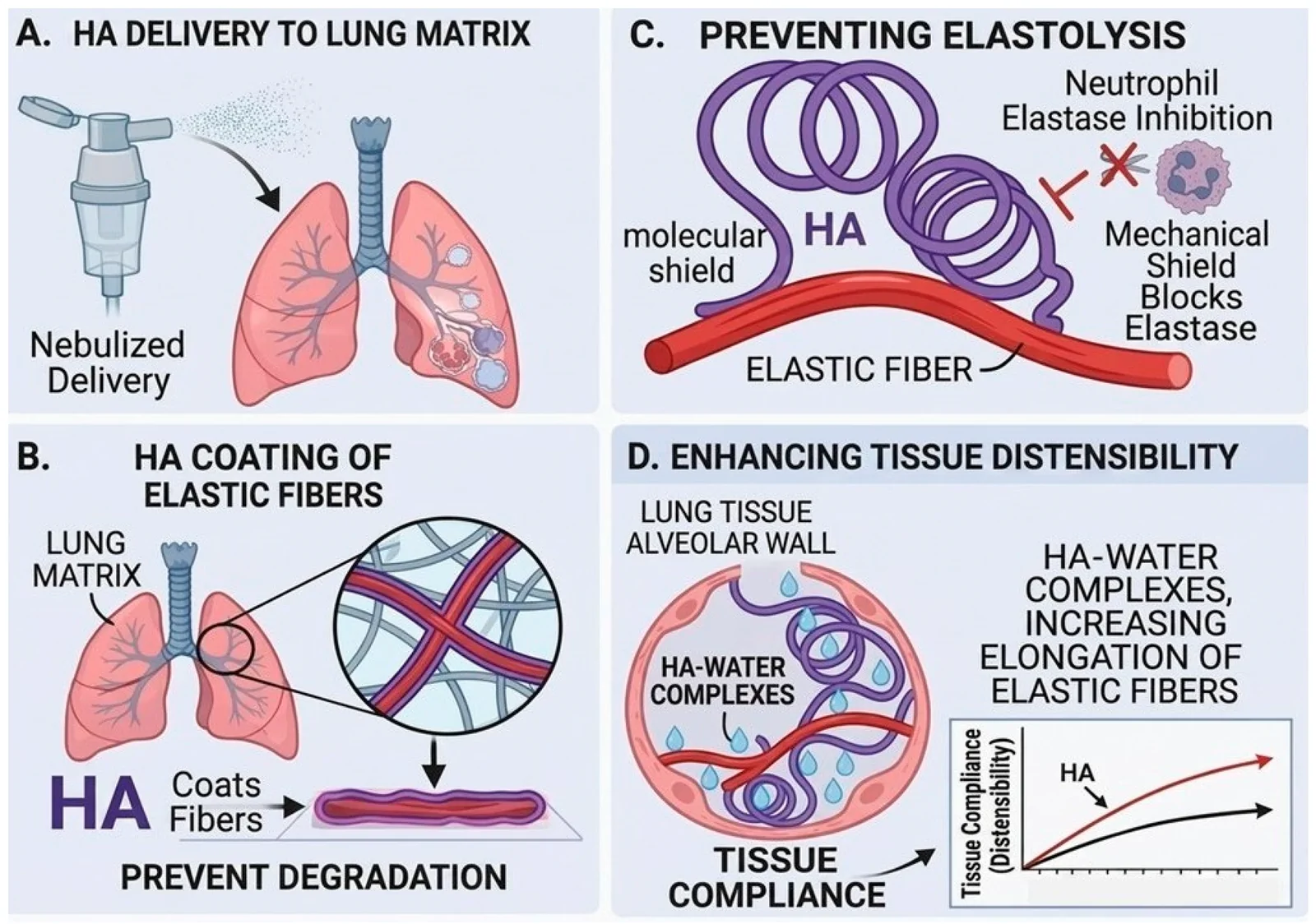
Inhaled HA (panel (**A**)) may have multiscale therapeutic effects, including protection of elastic fibers (panels (**B**,**C**)) from enzymatic degradation and improving airway mechanical properties (panel (**D**)).

Although this specific protective mechanism has not yet been demonstrated in CF patients or in CF-specific model systems, it is proposed that HMW-HA can similarly bind to elastic fiber surfaces in CF patients, forming a hydrating, steric barrier that reduces enzymatic access and mechanical abrasion, thereby helping preserve fiber extensibility and recoil properties (Figure 10). Whether this translates into stabilized airway mechanics in CF patients under chronic inflammatory stress remains to be established, and at this stage should be regarded as a biologically plausible hypothesis extrapolated from non-CF preclinical models rather than a demonstrated clinical benefit.

HA also exerts anti-inflammatory effects through interactions with CD44 receptors and suppression of NF-κB signaling, a regulatory axis well described for HA across chronic inflammatory tissue states [98]. This is particularly relevant in CF, where neutrophilic inflammation persists even in patients receiving highly effective CFTR modulators. HA’s ability to dampen excessive inflammatory signaling without immunosuppression may help slow the cycle of injury and remodeling. Additionally, HA forms a lubricating surface layer that protects airway epithelial cells from mechanical abrasion, limits bacterial adherence, and supports epithelial repair.

## 10. Animal Models and Translational Insights

Animal models have provided an advanced understanding of elastic fiber biology in CF, though each recapitulates only a subset of the human disease phenotype. The CFTR-knockout pig, developed to overcome the lack of spontaneous lung disease in CFTR-knockout mice, develops bronchiectasis, air trapping, and elastic fiber abnormalities in a timeline that approximates human CF, making it a valuable model for mechanistic and therapeutic studies [99]. The ferret CF model similarly develops spontaneous lung disease and has been used to study the temporal relationship between CFTR dysfunction, neutrophilic inflammation, and elastic fiber degradation [100].

Elastase-instillation models in rodents, while not specific to CF, have provided mechanistic insights into the consequences of unopposed NE or MMP-12 activity on lung elastic fiber architecture and function, informing the interpretation of clinical biomarker and intervention studies [14]. Conditional knockout models targeting specific elastic fiber components (ELN, FBN1, and FBLN5) in airway cells have clarified the relative contributions of these components to airway mechanics and susceptibility to inflammatory remodeling [18].

## 11. Pediatric Considerations: Elastic Fiber Development and Early CF

Elastic fiber assembly in the lung occurs primarily during fetal and early postnatal development, with the major period of alveolarization occurring in the first years of life. This developmental window coincides with a period of potential intense inflammation in CF infants, who develop airway infection and neutrophilic inflammation in the first months of life, even before symptoms become clinically apparent [35,101].

Bronchoalveolar lavage studies of CF infants have directly demonstrated elevated NE and MMP-9 concentrations in the first year of life, before the establishment of chronic Pseudomonas infection, suggesting that even Staphylococcus-driven or sterile inflammation may be sufficient to initiate elastic fiber degradation during the critical window of postnatal alveolarization [102]. However, it remains to be determined whether this early proteolytic burden accounts for the lung function decline seen in the first years of life [103].

## 12. Elastic Fibers, Inflammation, and the Vicious Cycle

A unifying concept that emerges from the preceding sections is that elastic fiber pathology in CF is not a passive downstream consequence of inflammation but an active participant in a self-amplifying pathological cycle. Elastic fiber degradation generates bioactive peptides (EDPs) that stimulate the recruitment of inflammatory cells and the production of mediators [12,18]. These inflammatory mediators drive further protease release and antiprotease inactivation [104]. Proteases degrade elastic fibers, releasing more EDPs, impairing mucociliary clearance by disrupting airway mechanics, and promoting mucus hypersecretion [18]. Accumulated mucus supports microbial growth, which drives further neutrophil influx and protease release.

Moreover, the fibrotic remodeling that accompanies and follows elastic fiber loss alters the biomechanical environment of surviving cells, thereby perpetuating the maladaptive response: stiffer, fibrotic tissue activates mechanosensing pathways in airway epithelial cells and fibroblasts, promoting further inflammation and collagen deposition [12,105]. The loss of elastic recoil alters the mechanical loading on airway smooth muscle, potentially contributing to airway hyperresponsiveness observed in a subset of CF patients. Understanding and interrupting this vicious cycle involving multiple processes, including CFTR function, protease/antiprotease balance, oxidative stress, EDP signaling, fibroblast behavior, and biomechanical environment, will likely be necessary to achieve meaningful structural preservation in CF.

## 13. Future Directions and Research Priorities

Several important questions remain to be addressed in order to fully exploit the therapeutic potential of targeting elastic fiber biology in CF. First, the relative contributions of NE, MMPs, cathepsins, and physicochemical mechanisms (oxidation, glycation) to net elastic fiber loss in vivo at different stages of CF lung disease require more precise quantification, ideally using isotope labeling or other in vivo turnover methods, building on classical isotope-based approaches to elastin turnover measurement [16]. Furthermore, the spatial heterogeneity of elastic fiber loss within the CF lung, which correlates poorly with lobar anatomy but closely with patterns of mucus obstruction and infection, needs to be mapped with higher resolution to identify the most vulnerable sites for targeted intervention [106].

The impact of CFTR modulators on elastic fiber biology in longitudinal patient cohorts, using desmosine and other biomarkers as endpoints, will be essential for determining whether current best therapies are sufficient to halt elastic fiber loss or whether adjunctive ECM-protective strategies will be necessary [77]. At the systemic level, the contributions from circulatory proteases, AGE formation, and nutritional deficiencies (copper, vitamin C) that impair LOX activity and antioxidant defenses deserves greater attention as an aspect of CF that may be amenable to treatment modalities other than inhaled interventions.

Finally, the application of single-cell transcriptomics and spatial proteomics to CF lung tissue will likely reveal novel cell-type-specific contributions to elastic fiber remodeling, potentially identifying new therapeutic targets among the diverse mesenchymal, immune, and epithelial cell populations that interact within the remodeling CF airway wall, including the CFTR-rich pulmonary ionocyte identified by recent single-cell atlases of the airway epithelium [107,108,109,110].

## 14. Conclusions

Elastic fiber degradation is a pervasive and mechanistically central feature of CF lung disease that extends from the molecular scale—where NE, MMPs, cathepsins, and oxidative species attack tropoelastin, fibrillin, and associated glycoproteins—to the organ scale, where the progressive loss of elastic tissue culminates in bronchiectasis, emphysema-like changes, impaired mucociliary clearance, and pulmonary hypertension. The irreversible nature of elastic fiber loss in adult tissues means that prevention rather than restoration must be the primary goal of therapeutic strategies targeting this component of CF pathology.

The transformative impact of CFTR modulators on the CF inflammatory milieu offers genuine hope that the cascade of events leading from CFTR dysfunction to elastic fiber degradation can be interrupted early and durably. However, the persistence of structural lung disease in modulator-treated patients and the insidious early-onset elastic fiber injury in CF infants underscore that CFTR correction alone may be insufficient to fully protect the elastic fiber network. Adjunctive strategies targeting protease/antiprotease balance, oxidative stress, and biosynthetic repair pathways will likely form part of a comprehensive therapeutic approach to preserving the structural integrity of CF airways in the coming era.

Ultimately, a deeper understanding of elastic fiber biology in CF—informed by biomarker-guided clinical studies, advances in animal models, and the application of modern omics technologies to CF tissue—will be essential to realizing the goal of a structurally normal lung in individuals living with CF.

## Figures and Tables

**Table 1:** Research Strategies Used for This Review.

Aspect	Description
Review Type	Narrative review rather than a systematic review.
Databases Searched	PubMed/MEDLINE.
Search Terms	Combinations of the terms: cystic fibrosis, elastic fiber, elastin, elastase, matrix metalloproteinase, extracellular matrix, bronchiectasis, CFTR modulator, and percolation theory.
Additional Search Methods	Hand-searching the reference lists of identified articles and key reviews.
Search Period	Through early 2026.
Study Selection Priority	Original mechanistic and clinical studies directly involving cystic fibrosis (CF) patients or validated CF model systems.
Contextual Literature Included	Foundational studies on elastic fiber biology and mechanistically informative studies from other lung diseases, such as emphysema and pulmonary fibrosis.
Purpose of Contextual Literature	To provide context when CF-specific data were unavailable.
Treatment of Extrapolated Findings	Findings extrapolated from non-CF studies were explicitly identified as such throughout the text.

## Data Availability

No new data were created or analyzed in this study. Data sharing is not applicable to this article.

## References

[1] Andersen D.H. (1938). Cystic fibrosis of the pancreas and its relation to celiac disease: A clinical and pathological study. Am. J. Dis. Child..

[2] Davis P.B. (2006). Cystic fibrosis since 1938. Am. J. Respir. Crit. Care Med..

[3] Riordan J.R., Rommens J.M., Kerem B., Alon N., Rozmahel R., Grzelczak Z., Zielenski J., Lok S., Plavsic N., Chou J.L. (1989). Identification of the cystic fibrosis gene: Cloning and characterization of complementary DNA. Science.

[4] Zielenski J., Tsui L.C. (1995). Cystic fibrosis: Genotypic and phenotypic variations. Annu. Rev. Genet..

[5] Cutting G.R. (2015). Cystic fibrosis genetics: From molecular understanding to clinical application. Nat. Rev. Genet..

[6] Ratjen F., Bell S.C., Rowe S.M., Goss C.H., Quittner A.L., Bush A. (2015). Cystic fibrosis. Nat. Rev. Dis. Primers.

[7] Rowe S.M., Miller S., Sorscher E.J. (2005). Cystic fibrosis. N. Engl. J. Med..

[8] O’Sullivan B.P., Freedman S.D. (2009). Cystic fibrosis. Lancet.

[9] Elborn J.S. (2016). Cystic fibrosis. Lancet.

[10] Chmiel J.F., Davis P.B. (2003). State of the art: Why do the lungs of patients with cystic fibrosis become infected, and why can’t they clear the infection?. Respir. Res..

[11] Flume P.A., O’Sullivan B.P., Robinson K.A., Goss C.H., Mogayzel P.J., Willey-Courand D.B., Bujan J., Finder J., Lester M., Quittell L. (2007). Cystic fibrosis pulmonary guidelines: Chronic medications for maintenance of lung health. Am. J. Respir. Crit. Care Med..

[12] Schmelzer C.E.H., Duca L. (2022). Elastic fibers: Formation, function, and fate during aging and disease. FEBS J..

[13] Durieu I., Peyrol S., Gindre D., Bellon G., Durand D.V., Pacheco Y. (1998). Subepithelial fibrosis and degradation of the bronchial extracellular matrix in cystic fibrosis. Am. J. Respir. Crit. Care Med..

[14] Voynow J.A., Shinbashi M. (2021). Neutrophil Elastase and Chronic Lung Disease. Biomolecules.

[15] Mall M.A., Davies J.C., Donaldson S.H., Jain R., Chalmers J.D., Shteinberg M. (2024). Neutrophil serine proteases in cystic fibrosis: Role in disease pathogenesis and rationale as a therapeutic target. Eur. Respir. Rev..

[16] Shapiro S.D., Endicott S.K., Province M.A., Pierce J.A., Campbell E.J. (1991). Marked longevity of human lung parenchymal elastic fibers deduced from prevalence of D-aspartate and nuclear weapons-related radiocarbon. J. Clin. Investig..

[17] Heinz A. (2020). Elastases and elastokines: Elastin degradation and its significance in health and disease. Crit. Rev. Biochem. Mol. Biol..

[18] Griese M., Kappler M., Gaggar A., Hartl D. (2008). Inhibition of airway proteases in cystic fibrosis lung disease. Eur. Respir. J..

[19] Ozsvar J., Yang C., Cain S.A., Baldock C., Tarakanova A., Weiss A.S. (2021). Tropoelastin and Elastin Assembly. Front. Bioeng. Biotechnol..

[20] Broekelmann T.J., Kozel B.A., Ishibashi H., Werneck C.C., Keeley F.W., Zhang L., Mecham R.P. (2005). Tropoelastin interacts with cell-surface glycosaminoglycans via its COOH-terminal domain. J. Biol. Chem..

[21] Schmelzer C.E.H., Heinz A., Troilo H., Lockhart-Cairns M.P., Jowitt T.A., Marchand M.F., Bidault L., Bignon M., Hedtke T., Barret A. (2019). Lysyl oxidase-like 2 (LOXL2)-mediated cross-linking of tropoelastin. FASEB J..

[22] Nakamura T., Lozano P.R., Ikeda Y., Iwanaga Y., Hinek A., Minamisawa S., Cheng C.-F., Kobuke K., Dalton N., Takada Y. (2002). Fibulin-5/DANCE is essential for elastogenesis in vivo. Nature.

[23] Yanagisawa H., Davis E.C., Starcher B.C., Ouchi T., Yanagisawa M., Richardson J.A., Olson E.N. (2002). Fibulin-5 is an elastin-binding protein essential for elastic fibre development in vivo. Nature.

[24] Horiguchi M., Inoue T., Ohbayashi T., Hirai M., Noda K., Marmorstein L.Y., Yabe D., Takagi K., Akama T.O., Kita T. (2009). Fibulin-4 conducts proper elastogenesis via interaction with cross-linking enzyme lysyl oxidase. Proc. Natl. Acad. Sci. USA.

[25] Choudhury R., McGovern A., Ridley C., Cain S.A., Baldwin A., Wang M.-C., Guo C., Mironov A., Drymoussi Z., Trump D. (2009). Differential regulation of elastic fiber formation by fibulin-4 and -5. J. Biol. Chem..

[26] Duca L., Floquet N., Alix A.J., Haye B., Debelle L. (2004). Elastin as a matrikine. Crit. Rev. Oncol. Hematol..

[27] Antonicelli F., Bellon G., Debelle L., Hornebeck W. (2007). Elastin-elastases and inflamm-aging. Curr. Top. Dev. Biol..

[28] Welsh M.J., Smith A.E. (1993). Molecular mechanisms of CFTR chloride channel dysfunction in cystic fibrosis. Cell.

[29] Cheng S.H., Gregory R.J., Marshall J., Paul S., Souza D.W., White G.A., O’Riordan C.R., Smith A.E. (1990). Defective intracellular transport and processing of CFTR is the molecular basis of most cystic fibrosis. Cell.

[30] Rogan M.P., Stoltz D.A., Hornick D.B. (2011). Cystic fibrosis transmembrane conductance regulator intracellular processing, trafficking, and opportunities for mutation-specific treatment. Chest.

[31] Boucher R.C. (2007). Airway surface dehydration in cystic fibrosis: Pathogenesis and therapy. Annu. Rev. Med..

[32] Matsui H., Grubb B.R., Tarran R., Randell S.H., Gatzy J.T., Davis C.W., Boucher R.C. (1998). Evidence for periciliary liquid layer depletion, not abnormal ion composition, in the pathogenesis of cystic fibrosis airways disease. Cell.

[33] Zabner J., Smith J.J., Karp P.H., Widdicombe J.H., Welsh M.J. (1998). Loss of CFTR chloride channels alters salt absorption by cystic fibrosis airway epithelia in vitro. Mol. Cell.

[34] Pilewski J.M., Frizzell R.A. (1999). Role of CFTR in airway disease. Physiol. Rev..

[35] Konstan M.W., Berger M. (1997). Current understanding of the inflammatory process in cystic fibrosis: Onset and etiology. Pediatr. Pulmonol..

[36] Chmiel J.F., Berger M., Konstan M.W. (2002). The role of inflammation in the pathophysiology of CF lung disease. Clin. Rev. Allergy Immunol..

[37] Birrer P., McElvaney N.G., Rüdeberg A., Sommer C.W., Liechti-Gallati S., Kraemer R., Hubbard R., Crystal R.G. (1994). Protease-antiprotease imbalance in the lungs of children with cystic fibrosis. Am. J. Respir. Crit. Care Med..

[38] Suter S., Chevallier I. (1991). Proteolytic inactivation of alpha 1-proteinase inhibitor in infected bronchial secretions from patients with cystic fibrosis. Eur. Respir. J..

[39] McElvaney N., Hubbard R., Birrer P., Crystal R., Chernick M., Frank M., Caplan D. (1991). Aerosol alpha 1-antitrypsin treatment for cystic fibrosis. Lancet.

[40] Taggart C., Coakley R.J., Greally P., Canny G., O’Neill S.J., McElvaney N.G. (2000). Increased elastase release by CF neutrophils is mediated by tumor necrosis factor-alpha and interleukin-8. Am. J. Physiol. Lung Cell. Mol. Physiol..

[41] Bergin D.A., Reeves E.P., Meleady P., Henry M., McElvaney O.J., Carroll T.P., Condron C., Chotirmall S.H., Clynes M., O’Neill S.J. (2010). Alpha-1 antitrypsin regulates human neutrophil chemotaxis induced by soluble immune complexes and IL-8. J. Clin. Investig..

[42] Gaggar A., Li Y., Weathington N., Winkler M., Kong M., Jackson P., Blalock J.E., Clancy J.P. (2007). Matrix metalloprotease-9 dysregulation in lower airway secretions of cystic fibrosis patients. Am. J. Physiol. Lung Cell. Mol. Physiol..

[43] Trojanek J.B., Cobos-Correa A., Diemer S., Kormann M., Schubert S.C., Zhou-Suckow Z., Agrawal R., Duerr J., Wagner C.J., Schatterny J. (2014). Airway mucus obstruction triggers macrophage activation and matrix metalloproteinase 12-dependent emphysema. Am. J. Respir. Cell. Mol. Biol..

[44] Sepper R., Konttinen Y.T., Ding Y., Takagi M., Sorsa T. (1995). Human neutrophil collagenase (MMP-8), identified in bronchiectasis BAL fluid, correlates with severity of disease. Chest.

[45] Prikk K., Maisi P., Pirilä E., Reintam M.-A., Salo T., Sorsa T., Sepper R. (2002). Airway obstruction correlates with collagenase-2 (MMP-8) expression and activation in bronchial asthma. Lab. Investig..

[46] Ratjen F., Hartog C.M., Paul K., Wermelt J., Braun J. (2002). Matrix metalloproteases in BAL fluid of patients with cystic fibrosis and their modulation by treatment with dornase alpha. Thorax.

[47] Heinz A., Jung M.C., Duca L., Sippl W., Taddese S., Ihling C., Rusciani A., Jahreis G., Weiss A.S., Neubert R.H.H. (2010). Degradation of tropoelastin by matrix metalloproteinases-cleavage site specificities and release of matrikines. FEBS J..

[48] Hilliard T.N., Regamey N., Shute J.K., Nicholson A.G., Alton E.W.F.W., Bush A., Davies J.C. (2007). Airway remodelling in children with cystic fibrosis. Thorax.

[49] Meyts I., Gutiérrez M.J., Doherty T.A., Almeida C., Khader S., Lammi W., De Boeck C. (2021). AGE-RAGE signaling in cystic fibrosis. Pediatr. Pulmonol..

[50] Vlassara H., Uribarri J. (2014). Advanced glycation end products (AGE) and diabetes: Cause, effect, or both?. Curr. Diabetes Rep..

[51] Konova E., Baydanoff S., Atanasova M., Velkova A. (2004). Age-related changes in the glycation of human aortic elastin. Exp. Gerontol..

[52] Rucker R.B., Kosonen T., Clegg M.S., Mitchell A.E., Rucker B.R., Uriu-Hare J.Y., Keen C.L. (1998). Copper, lysyl oxidase, and extracellular matrix protein cross-linking. Am. J. Clin. Nutr..

[53] Harris W.T., Kelly D.R., Zhou Y., Wang D., Macewen M., Hagood J.S., Clancy J.P., Ambalavanan N., Sorscher E.J. (2013). Myofibroblast differentiation and enhanced TGF-beta signaling in cystic fibrosis lung disease. PLoS ONE.

[54] Sly P.D., Gangell C.L., Chen L., Ware R., Ranganathan S., Mott L.S., Murray C.P., Stick S. (2013). Risk factors for bronchiectasis in children with cystic fibrosis. N. Engl. J. Med..

[55] Hayes D., Tobias J.D., Mansour H.M., Kirkby S. (2014). Pulmonary hypertension in cystic fibrosis. Lung.

[56] Thenappan T., Chan S.Y., Weir E.K. (2018). Role of extracellular matrix in the pathogenesis of pulmonary arterial hypertension. Am. J. Physiol. Heart Circ. Physiol..

[57] Sherwood J.S., Ullal J., Kutney K., Hughan K.S. (2022). Cystic fibrosis related liver disease and endocrine considerations. J. Clin. Transl. Endocrinol..

[58] Debray D., Kelly D., Houwen R., Strandvik B., Colombo C. (2011). Best practice guidance for the diagnosis and management of cystic fibrosis-associated liver disease. J. Cyst. Fibros..

[59] Fiorotto R., Strazzabosco M. (2019). Pathophysiology of cystic fibrosis liver disease: A channelopathy leading to alterations in innate immunity and in microbiota. Cell Mol. Gastroenterol. Hepatol..

[60] Assis D.N., Debray D. (2017). Gallbladder and bile duct disease in cystic fibrosis. J. Cyst. Fibros..

[61] Ma S., Geraghty P., Dabo A., McCarthy C., McElvaney N.G., Turino G.M. (2019). Cystic fibrosis disease severity correlates with plasma levels of desmosine and isodesmosine, biomarkers of elastin degradation. ERJ Open Res..

[62] Viglio S., Iadarola P., Lupi A., Trisolini R., Tinelli C., Balbi B., Grassi V., Worlitzsch D., Döring G., Meloni F. (2000). MEKC of desmosine and isodesmosine in urine of chronic destructive lung disease patients. Eur. Respir. J..

[63] Devenport N.A., Reynolds J.C., Parkash V., Cook J., Weston D.J., Creaser C.S. (2011). Determination of free desmosine and isodesmosine as urinary biomarkers of lung disorder using ultra performance liquid chromatography-ion mobility-mass spectrometry. J. Chromatogr. B Anal. Technol. Biomed. Life Sci..

[64] Albarbarawi O., Barton A., Miller D., McSharry C., Chaudhuri R., Thomson N.C., NA Palmer C., Devereux G., Huang J.T.-J. (2013). Characterization and validation of an isotope-dilution LC-MS/MS method for quantification of total desmosine and isodesmosine in plasma and serum. Bioanalysis.

[65] Turino G.M., Ma S., Lin Y.Y., Cantor J.O., Luisetti M. (2011). Matrix elastin: A promising biomarker for chronic obstructive pulmonary disease. Am. J. Respir. Crit. Care Med..

[66] Turino G.M., Ma S., Cantor J.O., Lin Y.Y. (2016). Biomarkers in alpha-1 antitrypsin deficiency chronic obstructive pulmonary disease. Ann. Am. Thorac. Soc..

[67] Laguna T.A., Wagner B.D., Luckey H.K., Mann S.A., Sagel S.D., Regelmann W., Accurso F.J. (2009). Sputum desmosine during hospital admission for pulmonary exacerbation in cystic fibrosis. Chest.

[68] McElvaney O.J., Gunaratnam C., Reeves E.P., McElvaney N.G. (2019). A specialized method of sputum collection and processing for therapeutic interventions in cystic fibrosis. J. Cyst. Fibros. Off. J. Eur. Cyst. Fibros. Soc..

[69] Ramsey B.W., Davies J., McElvaney N.G., Tullis E., Bell S.C., Dřevínek P., Griese M., McKone E.F., Wainwright C.E., Konstan M.W. (2011). A CFTR potentiator in patients with cystic fibrosis and the G551D mutation. N. Engl. J. Med..

[70] Accurso F.J., Rowe S.M., Clancy J.P., Boyle M.P., Dunitz J.M., Durie P.R., Sagel S.D., Hornick D.B., Konstan M.W., Donaldson S.H. (2010). Effect of VX-770 in persons with cystic fibrosis and the G551D-CFTR mutation. N. Engl. J. Med..

[71] Chmiel J.F., Konstan M.W., Elborn J.S. (2013). Antibiotic and anti-inflammatory therapies for cystic fibrosis. Cold Spring Harb. Perspect. Med..

[72] Heijerman H.G.M., McKone E.F., Downey D.G., Van Braeckel E., Rowe S.M., Tullis E., Mall M.A., Welter J.J., Ramsey B.W., McKee C.M. (2019). Efficacy and safety of the elexacaftor plus tezacaftor plus ivacaftor combination regimen in people with cystic fibrosis homozygous for the F508del mutation. Lancet.

[73] Middleton P.G., Mall M.A., Dřevínek P., Lands L.C., McKone E.F., Polineni D., Ramsey B.W., Taylor-Cousar J.L., Tullis E., Vermeulen F. (2019). Elexacaftor-tezacaftor-ivacaftor for cystic fibrosis with a single Phe508del allele. N. Engl. J. Med..

[74] Zemanick E.T., Taylor-Cousar J.L., Davies J., Gibson R.L., Mall M.A., McKone E.F., McNally P., Ramsey B.W., Rayment J.H., Rowe S.M. (2021). A phase 3 open-label study of elexacaftor/tezacaftor/ivacaftor in children 6 through 11 years of age with cystic fibrosis and at least one F508del allele. Am. J. Respir. Crit. Care Med..

[75] Wainwright C.E., Elborn J.S., Ramsey B.W., Marigowda G., Huang X., Cipolli M., Colombo C., Davies J.C., De Boeck K., Flume P.A. (2015). Lumacaftor-ivacaftor in patients with cystic fibrosis homozygous for Phe508del CFTR. N. Engl. J. Med..

[76] Stoltz D.A., Meyerholz D.K., Welsh M.J. (2015). Origins of cystic fibrosis lung disease. N. Engl. J. Med..

[77] Mall M.A., Mayer-Hamblett N., Rowe S.M. (2020). Cystic fibrosis: Emergence of highly effective targeted therapeutics and potential clinical implications. Am. J. Respir. Crit. Care Med..

[78] Petridou N.I., Corominas-Murtra B., Heisenberg C.P., Hannezo E. (2021). Rigidity percolation uncovers a structural basis for embryonic tissue phase transitions. Cell.

[79] Elkins M.R., Robinson M., Rose B.R., Harbour C., Moriarty C.P., Marks G.B., Belousova E.G., Xuan W., Bye P.T. (2006). A controlled trial of long-term inhaled hypertonic saline in patients with cystic fibrosis. N. Engl. J. Med..

[80] Ratjen F. (2006). Restoring airway surface liquid in cystic fibrosis. N. Engl. J. Med..

[81] Cohen T.S., Prince A. (2012). Cystic fibrosis: A mucosal immunodeficiency syndrome. Nat. Med..

[82] Xu X., Jackson P.L., Tanner S., Hardison M.T., Roda M.A., Blalock J.E., Gaggar A. (2011). A self-propagating matrix metalloprotease-9 (MMP-9) dependent cycle of chronic neutrophilic inflammation. PLoS ONE.

[83] Scandolera A., Odoul L., Salesse S., Guillot A., Blaise S., Kawecki C., Maurice P., El Btaouri H., Romier-Crouzet B., Martiny L. (2016). The elastin receptor complex: A unique matricellular receptor with high anti-tumoral potential. Front. Pharmacol..

[84] Wahart A., Hocine T., Albrecht C., Henry A., Sarazin T., Martiny L., El Btaouri H., Maurice P., Bennasroune A., Romier-Crouzet B. (2019). Role of elastin peptides and elastin receptor complex in metabolic and cardiovascular diseases. FEBS J..

[85] Hubbard R.C., Fells G., Gadek J., Pacholok S., Humes J., Crystal R.G. (1991). Neutrophil accumulation in the lung in alpha-1-antitrypsin deficiency: Spontaneous release of leukotriene B4 by alveolar macrophages. J. Clin. Investig..

[86] Griese M., Latzin P., Kappler M., Weckerle K., Heinzlmaier T., Bernhardt T., Hartl D. (2007). Alpha1-antitrypsin inhalation reduces airway inflammation in cystic fibrosis patients. Eur. Respir. J..

[87] Stevens T., Ekholm K., Gränse M., Lindahl M., Kozma V., Jungar C., Ottosson T., Falk-Håkansson H., Churg A., Wright J.L. (2011). AZD9668: Pharmacological characterization of a novel oral inhibitor of neutrophil elastase. J. Pharmacol. Exp. Ther..

[88] Xu X., Abdalla T., Bratcher P.E., Jackson P.L., Sabbatini G., Wells J.M., Lou X.-Y., Quinn R., Blalock J.E., Clancy J.P. (2017). Doxycycline improves clinical outcomes during cystic fibrosis exacerbations. Eur. Respir. J..

[89] Griese M., Kappler M., Eismann C., Ballmann M., Junge S., Rietschel E., van Koningsbruggen-Rietschel S., Staab D., Rolinck-Werninghaus C., Mellies U. (2013). Inhalation treatment with glutathione in patients with cystic fibrosis: A randomized clinical trial. Am. J. Respir. Crit. Care Med..

[90] Kauffman K.J., Webber M.J., Anderson D.G. (2016). Materials for non-viral intracellular delivery of messenger RNA therapeutics. J. Control. Release.

[91] Robinson E., MacDonald K.D., Slaughter K., McKinney M., Patel S., Sun C., Sahay G. (2018). Lipid nanoparticle-delivered chemically modified mRNA restores chloride secretion in cystic fibrosis. Mol. Ther..

[92] Firth A.L., Dargitz C.T., Qualls S.J., Menon T., Wright R., Singer O., Gage F.H., Khanna A., Verma I.M. (2014). Generation of multiciliated cells in functional airway epithelia from human induced pluripotent stem cells. Proc. Natl. Acad. Sci. USA.

[93] Jiang D., Liang J., Noble P.W. (2007). Hyaluronan in tissue injury and repair. Annu. Rev. Cell Dev. Biol..

[94] Aya K.L., Stern R. (2014). Hyaluronan in wound healing: Rediscovering a major player. Wound Repair Regen..

[95] Stern R., Kogan G., Jedrzejas M.J., Soltes L. (2007). The many ways to cleave hyaluronan. Biotechnol. Adv..

[96] Stauffer B.L., Ihnat M.A., Miyahira A.K., Patel R., Witte D.P., Brown D.M., Chittur K.K. (2003). Hyaluronan biosynthesis diminishes shear-induced fluid pressure. Biorheology.

[97] Cantor J.O., Shteyngart B., Cerreta J.M., Liu M., Armand G., Turino G.M. (2000). The effect of hyaluronan on elastic fiber injury in vitro and elastase-induced airspace enlargement in vivo. Proc. Soc. Exp. Biol. Med..

[98] Chen Y., Peng Y., Zhu W., Li J., Wu M., Guo Y., Li Y., Wang X., Li X. (2019). Hyaluronic acid modulates airway epithelial and inflammatory responses: Implications for chronic airway disease therapeutics. Mediat. Inflamm..

[99] Stoltz D.A., Meyerholz D.K., Pezzulo A.A., Ramachandran S., Rogan M.P., Davis G.J., Hanfland R.A., Wohlford-Lenane C., Dohrn C.L., Bartlett J.A. (2010). Cystic fibrosis pigs develop lung disease and exhibit defective bacterial eradication at birth. Sci. Transl. Med..

[100] Sun X., Olivier A.K., Liang B., Yi Y., Sui H., Evans T.I.A., Zhang Y., Zhou W., Tyler S.R., Fisher J.T. (2014). Lung phenotype of juvenile and adult cystic fibrosis transmembrane conductance regulator-knockout ferrets. Am. J. Respir. Cell Mol. Biol..

[101] Khan T.Z., Wagener J.S., Bost T., Martinez J., Accurso F.J., Riches D.W. (1995). Early pulmonary inflammation in infants with cystic fibrosis. Am. J. Respir. Crit. Care Med..

[102] Gangell C., Gard S., Douglas T., Park J., de Klerk N., Keil T., Brennan S., Ranganathan S., Robins-Browne R., Sly P.D. (2011). Inflammatory responses to individual microorganisms in the lungs of children with cystic fibrosis. Clin. Infect. Dis..

[103] Mott L.S., Park J., Murray C.P., Gangell C.L., de Klerk N.H., Robinson P.J., Robertson C.F., Ranganathan S.C., Sly P.D., Stick S.M. (2012). Progression of early structural lung disease in young children with cystic fibrosis assessed using CT. Thorax.

[104] Cohen-Cymberknoh M., Kerem E., Ferkol T., Elizur A. (2013). Airway inflammation in cystic fibrosis: Molecular mechanisms and clinical implications. Thorax.

[105] Cantin A.M., Hartl D., Konstan M.W., Chmiel J.F. (2015). Inflammation in cystic fibrosis lung disease: Pathogenesis and therapy. J. Cyst. Fibros..

[106] De Jong P.A., Mayo J.R., Golmohammadi K., Nakano Y., Lequin M.H., Tiddens H.A.W.M., Aldrich J., Coxson H.O., Sin D.D. (2006). Estimation of cancer mortality associated with repetitive computed tomography scanning. Am. J. Respir. Crit. Care Med..

[107] Travaglini K.J., Nabhan A.N., Penland L., Sinha R., Gillich A., Sit R.V., Chang S., Conley S.D., Mori Y., Seita J. (2020). A molecular cell atlas of the human lung from single-cell RNA sequencing. Nature.

[108] Vieira Braga F.A., Kar G., Berg M., Carpaij O.A., Polanski K., Simon L.M., Brouwer S., Gomes T., Hesse L., Jiang J. (2019). A cellular census of human lungs identifies novel cell states in health and in asthma. Nat. Med..

[109] Plasschaert L.W., Žilionis R., Choo-Wing R., Savova V., Knehr J., Roma G., Klein A.M., Jaffe A.B. (2018). A single-cell atlas of the airway epithelium reveals the CFTR-rich pulmonary ionocyte. Nature.

[110] Montoro D.T., Haber A.L., Biton M., Vinarsky V., Lin B., Birket S.E., Yuan F., Chen S., Leung H.M., Villoria J. (2018). A revised airway epithelial hierarchy includes CFTR-expressing ionocytes. Nature.

